# Empagliflozin and Metformin Combination Therapy Attenuates Oxidative Stress and Inflammation in Experimental Cardio–Kidney–Metabolic Syndrome

**DOI:** 10.3390/ijms27208988

**Published:** 2026-10-10

**Authors:** Fernando Cortes-Camacho, Oscar R. Zambrano-Vásquez, Fernando E. García-Arroyo, Elena Aréchaga-Ocampo, Jorge I. Castañeda-Sánchez, Daniel Manzur-Sandoval, Francisco Javier Roldan-Gómez, Leonardo Del Valle-Mondragón, O. Emiliano Aparicio-Trejo, Constanza E. Martínez-Olivares, Rogelio Hernández-Pando, José G. Gonzaga-Sánchez, Laura G. Sánchez-Lozada, Horacio Osorio-Alonso

**Affiliations:** 1 Doctorado en Ciencias Biológicas y de la Salud, Universidad Autónoma Metropolitana-Xochimilco, Mexico City 04960, Mexico; fernando.cortes@cua.uam.mx (F.C.-C.); oscar.zambrano@cua.uam.mx (O.R.Z.-V.); 2 Departamento de Fisiopatología Cardio-Renal, Instituto Nacional de Cardiología Ignacio Chávez, Mexico City 14080, Mexico; enrique.garcia@cardiologia.org.mx (F.E.G.-A.); omar.aparicio@cardiologia.org.mx (O.E.A.-T.); jose.gonzaga@cardiologia.org.mx (J.G.G.-S.); laura.sanchez@cardiologia.org.mx (L.G.S.-L.); 3 Departamento de Ciencias Naturales, Universidad Autónoma Metropolitana, Unidad Cuajimalpa, Mexico City 05348, Mexico; earechaga@cua.uam.mx; 4 Departamento de Sistemas Biológicos, Universidad Autónoma Metropolitana, Unidad Xochimilco, Mexico City 04960, Mexico; jcastanedas@correo.xoc.uam.mx; 5 Cardiovascular Critical Care Unit, Instituto Nacional de Cardiología Ignacio Chávez, Mexico City 14080, Mexico; daniel.manzur@cardiologia.org.mx; 6 Outpatient Department, Instituto Nacional de Cardiología Ignacio Chávez, Mexico City 14080, Mexico; roldan@cardiologia.org.mx; 7 Pharmacology Department, Instituto Nacional de Cardiología Ignacio Chávez, Mexico City 14080, Mexico; leonardo.delvalle@cardiologia.org.mx; 8 Experimental Pathology Department, Instituto Nacional de Ciencia Médicas y Nutrición “Salvador Zubirán”, Mexico City 14080, Mexico; constanzamtz@exalumno.unam.mx (C.E.M.-O.); rogelio.hernandezp@incmnsz.mx (R.H.-P.)

**Keywords:** metabolic risk factors, cardiovascular risk, renal dysfunction, hyperuricemia, hypertension, oxidative stress, inflammation, empagliflozin, metformin

## Abstract

This study evaluated the therapeutic effects of the empagliflozin+metformin (E+M) combination on cardio–kidney–metabolic syndrome (CKMS) using an experimental model. Animals were fed a Paigen-type (high-fat) diet supplemented with sugar-sweetened beverages to induce the CKMS phenotype. After confirming metabolic, cardiovascular, and renal alterations, animals were randomized into untreated and E+M-treated groups for 30 days while continuing the diet. Cardiovascular risk factors, vasoactive substances in the heart and kidney, oxidative stress, and the expression of inflammatory markers and the lipogenic transcription factors ChREBP and SREBP1 were analyzed. The E+M combination reduced body weight, hyperglycemia, fasting glucose, and hypertension, while restoring the lipid profile and improving oral glucose tolerance and renal function (*p* < 0.05). It also reduced posterior wall and interventricular septal thickening, as well as the elevated septum/posterior wall (PP/PS) ratio observed in the CKMS group (*p* < 0.05). Likewise, the increases in angiotensin II and nitric oxide concentrations in the hearts and kidneys were attenuated (*p* < 0.05). The CKMS group exhibited glomerular hypertrophy and reduced total antioxidant capacity mitochondrial complex I and II activities, glutathione peroxidase, glutathione reductase, and superoxide dismutase activities, and Nrf2 and IL-10 expression, all of which were restored by combination therapy (*p* < 0.05). In contrast, the upregulation of IGF-1, aldose reductase, sorbitol dehydrogenase, ChREBP, IL-6, TNFα, and NF-κB observed in the CKMS group was reduced following E+M treatment (*p* < 0.05). Overall, the E+M combination attenuated metabolic alterations and suppressed renal oxidative stress and inflammation, providing cardiac and renal protection at both functional and structural levels. These effects appear to be mediated by the modulation of key proteins involved in vascular tone, uric acid metabolism, oxidative stress, inflammation, and regulation of transcription factors involved in carbohydrate and lipid metabolism.

## 1. Introduction

Cardio–kidney–metabolic syndrome (CKMS) is a health condition characterized by overlapping pathological features, including clinical conditions such as obesity and comorbidities such as hypertension, diabetes, metabolic syndrome (MetS), hyperlipidemia, and chronic kidney disease (CKD) [1,2]. In this context, MetS is a complex disorder characterized by central obesity, insulin resistance (IR), hyperglycemia, dyslipidemia, and hypertension [3]. MetS not only predisposes individuals to type 2 diabetes mellitus (T2DM), but also increases the risk of severe complications, particularly cardiovascular disease (CVD) [4], and CKD [5] both of which contribute to the development and progression of CKMS.

The pathogenesis of CKMS arises from a complex interplay of factors such as inflammation, oxidative stress, IR, lipotoxicity, among other factors, all of which progressively impair cardiac and renal function [1,6]. CKMS is associated with an increased risk of all-cause mortality (hazard ratios 1.33), cardiovascular mortality (HR: 2.81), and the incidence of end-stage kidney disease (HR: 10.15). Interestingly, the risk of adverse outcomes increases with the number of CKMS components present [2]. Each additional CKMS component is associated with a 22% increase in all-cause mortality risk (HR: 1.22; 95% CI: 1.21, 1.23), a 37% increase in CVD mortality risk (HR: 1.37; 95% CI: 1.35, 1.40) compared with individuals without CKMS components. Furthermore, each additional component is associated with an approximate reduction of three years in average life expectancy [2].

Independently, the metabolic abnormalities and risk factors activate pathological cellular mechanisms that can amplify or feedback on one another. For example, hyperuricemia (HU) [elevated uric acid (UA) levels] has been recognized as a risk factor that contributes to IR and to the development of hypertension, CVD, and CKD [7,8,9]. In addition, hyperuricemia promotes CVD by inducing left ventricular dysfunction and ischemic heart disease, as well as by causing renal damage due to systemic and glomerular hypertension, nitric oxide (NO) inhibition, oxidative stress, and inflammation [10,11].

On the other hand, IR causes postprandial hyperglycemia, which can activate aldose reductase (AR) and sorbitol dehydrogenase (SDH) [12,13]. Both enzymes contribute to oxidative stress because the conversion of glucose to sorbitol and sorbitol to fructose depletes nicotinamide adenine dinucleotide phosphate (NADPH), the limiting cofactor for the synthesis of reduced glutathione, one of the main endogenous antioxidants [13]. The resulting fructose is primarily metabolized by fructokinase in the liver and the kidney, leading to the depletion of adenosine triphosphate (ATP), resulting in adenosine monophosphate, which subsequently converts into UA [9]. Furthermore, MetS may lead to HU because of increased UA synthesis, impaired renal excretion, or both. Although UA can act as an antioxidant, it also poses risk to cardiac and renal health [14,15].

UA activates NADPH oxidase (NOX) and nuclear factor kappa B (NF-κB), resulting in decreased NO synthesis and bioavailability, endothelial damage, and the exacerbation of kidney damage and CVD [16]. Reactive oxygen species (ROS) can harm mitochondrial membranes, thereby impairing their bioenergetic function and ATP production [17]. Moreover, sorbitol, an intermediate metabolite of this pathway, accumulates within cells, leading to osmotic damage that has been linked to the onset of CKD [18].

Additionally, IR and hypertension, key components of MetS, can further impair kidney function by increasing oxidative stress and triggering pro-inflammatory and hemodynamic pathways, such as the renin angiotensin aldosterone system (RAAS), which is well known to harm blood vessels as well as the brain, heart, and kidneys [19]. Furthermore, inflammation has been described as a key factor involved in the progression of CKMS in patients and experimental models [5,20], where increased levels of C-reactive protein (CRP), Interleukin-1 beta (IL-1β), Interleukin-6 (IL-6), and tumor necrosis factor-alpha (TNFα), or the activation of nuclear factor kappa B (NF-κB), and nucleotide-binding domain, leucine-rich repeat, and pyrin domain-containing protein 3 (NLRP3) pathways promote and amplify systemic damage, and in this specific case, damage to the heart and kidneys [5,21,22,23]. The consequences of CKMS impose a significant economic burden for public health institutions and patients. To reduce fatal outcomes and improve patient prognosis and quality of life, therapeutic strategies should address multiple risk factors while also lowering healthcare costs. In this study, we propose the use of a combination of empagliflozin/metformin for the treatment of CKMS.

Empagliflozin, classified as a sodium–glucose cotransporter type 2 inhibitor (SGLT2i) is recommended as a second- or third-line antidiabetic drug and has demonstrated protective effects on both renal and cardiovascular function [24]. In comparison, metformin remains the first-line treatment for diabetes. Additionally, metformin acts as an activator of AMP-activated protein kinase (AMPK), contributing to reduced renal oxidative stress and improved lipid metabolism [25,26]. On the other hand, in metabolic diseases, achieving strict metabolic control through monotherapy is difficult and often not possible; therefore, several studies suggest that the use of combination therapy in early stages will help improve long-term metabolic control and reduce the risk of associated complications [27,28]. Due to their distinct yet complementary mechanisms of action, the concurrent use of empagliflozin and metformin may provide synergistic protection against CKMS. Therefore, the present study seeks to evaluate the therapeutic effects of empagliflozin/metformin combination on CKMS, with particular emphasis on renal oxidative stress and inflammation, using a diet-induced experimental model.

## 2. Results

### 2.1. Characterization of the Metabolic and Cardiovascular Risk Factors, and Cardiac and Renal Function

#### 2.1.1. Determination of Metabolic Parameters

The dietary intervention successfully induced a state of metabolic dysfunction. The Paigen-type diet supplemented with a SSB led to pronounced cardiovascular risks factors and significant alterations in both cardiac and renal functional parameters in the CKMS group.

Specifically, the CKMS group exhibited a marked increase in body weight gain compared with the control group fed a maintenance diet (Table 1, *p* < 0.05 vs. control group). This was accompanied by a significant elevation in blood glucose concentrations (Table 1, *p* < 0.05 vs. control group) and severely impaired glucose metabolism, as confirmed by the oral glucose tolerance test (OGTT) (Table 1). Although baseline and fasting glucose levels remained comparable between groups, the CKMS group showed a significantly higher area under the curve (AUC) obtained following an oral glucose load (3 g/kg), compared with the controls (Table 1, *p* < 0.05 vs. control group). Notably, blood glucose levels in the CKMS group failed to return to baseline levels within the monitored period, indicating glucose intolerance. It is important to note that, as is typical for diet-induced metabolic syndrome models, fasting blood glucose levels showed a moderate elevation (106.4 ± 1.96 mg/dL vs. 87.80 ± 2.24 mg/dL in controls) rather than overt diabetic hyperglycemia, reflecting an insulin-resistant prediabetic state.

Similarly, the CKMS group exhibited significantly increased plasma concentrations of triglycerides and total cholesterol, along with reduced high-density lipoprotein cholesterol (HDL-c) levels, compared with the control group (Table 1, *p* < 0.05 vs. control group). Furthermore, a substantial increase in free fatty acid (FFA) concentration was observed in the CKMS group (Table 1, *p* < 0.05 vs. control group). These data confirm the establishment of metabolic alterations that define metabolic syndrome. Consequently, we further evaluated cardiovascular risk factors, including UA levels and blood pressure, and cardiac function.

#### 2.1.2. Cardiovascular Risk Factors and Cardiac Function

Table 1 illustrates the impact of the dietary intervention on plasma and urinary UA levels, systolic blood pressure (SBP), and cardiac function assessed by echocardiography. The CKMS group showed significantly higher plasma UA levels (Table 1, *p* < 0.05 vs. control group), indicating altered purine metabolism and the development of hyperuricemia. Additionally, the CKMS group demonstrated elevated SBP compared to the control group (Table 1, *p* < 0.05 vs. control group), suggestive of established hypertension.

Echocardiographic analysis was performed to determine whether the dietary intervention induced structural and functional cardiac alterations.

The results revealed that the CKMS group had a significant increase in the thickness of both the posterior wall (PWT) and the interventricular septum (IVS), alongside an increased PWT/IVS ratio, which is indicative of left ventricle hypertrophy (LVH) (Figure 1A,B,D). This wall thickening represents an adaptive response to increased cardiac workload. Furthermore, a significant reduction in left ventricular diastolic diameter (LVDD) was observed (Figure 1C, * *p* < 0.05 vs. control group), suggesting impaired relaxation and filling consistent with diastolic dysfunction. Finally, the significant increase in relative wall thickness (Figure 1D, *p* < 0.05 vs. control group) confirmed concentric remodeling of the left ventricle.

#### 2.1.3. Assessment of Renal Function

Given the strong association between metabolic dysfunction, cardiovascular risk, and renal impairment, we investigated whether this pathogenic cluster leads to kidney damage. Renal biomarkers [creatinine, blood urea nitrogen (BUN), and proteinuria] showed marked alterations in the CKMS group. Plasma creatinine and BUN levels were significantly elevated (Table 1, *p* < 0.05 vs. control group), indicating impaired renal clearance. Notably, proteinuria was significantly higher in the CKMS group, suggesting tubular and glomerular structural damage (Table 1, *p* < 0.05 vs. control group). Finally, a significant increase in diuresis was observed in the CKMS group (Table 1, *p* < 0.05 vs. control group).

In summary, 30 days of high-fat diet (HFD)/SSB intake led to the development of a complex phenotype characterized by metabolic, cardiovascular, and renal impairment. Taken together, these data confirm the successful establishment of CKMS. Therefore, this model was subsequently used to evaluate the therapeutic potential of the empagliflozin/metformin combination.

### 2.2. Effect of the Combination of Empagliflozin/Metformin on Cardio–Kidney–Metabolic Syndrome

#### 2.2.1. Impact of Empagliflozin/Metformin on Metabolic Alteration in CKMS Individuals

Thirty days of pharmacological treatment with the empagliflozin/metformin combination in the CKMS group demonstrated several beneficial effects.

The untreated CKMS group maintained pronounced metabolic derangements, including increased body weight and fasting glucose levels (Figure 2A,B, *p* < 0.05 vs. control group), as well as persistent glucose intolerance. This was evidenced by the OGTT, where a higher peak of glucose in the untreated CMKS group was observed at 30 min after oral glucose overload, which failed to return to baseline levels within the monitored period (150 min) (Figure 2C).

Additionally, an impaired lipid profile was observed in the CKMS group, characterized by elevated triglyceride and total cholesterol levels (Figure 2D,E, *p* < 0.05 vs. control group), along with a significant reduction in HDL-c levels compared to the control group (Figure 2F, *p* < 0.05 vs. control group). The CKMS group also maintained elevated FFA levels compared with the control group, indicating altered lipid metabolism characteristic of this condition (Figure 2G).

In contrast, treatment with the empagliflozin and metformin (E+M) combination effectively resulted in significant weight reduction, improved fasting glucose levels and glucose tolerance, and decreased plasma triglyceride, total cholesterol, and FFA levels compared to the CKMS group (Figure 2A–E,G, *p* < 0.05 vs. CKMS group). Additionally, it led to a significant increase in plasma HDL-c levels (Figure 2F, *p* < 0.05 vs. CKMS group), approaching values observed in the control group.

#### 2.2.2. Efficacy of Combined Empagliflozin and Metformin Therapy in Controlling Cardiovascular Risk Factors

After 30 days of treatment, the untreated CKMS group exhibited a sustained and significant increase in SBP compared to the control group (Figure 3A, *p* < 0.05 vs. control group). Additionally, the CKMS group demonstrated a marked elevation in plasma UA levels (Figure 3A, *p* < 0.05 vs. control group) and a concomitant increase in urinary UA excretion, confirming the presence of hyperuricemia.

In contrast, the combined treatment with E+M proved highly effective in mitigating these risk factors. E+M therapy restored both UA levels and SBP to values comparable to those of the control group (Figure 3A,B, *p* < 0.05 vs. CKMS group). Furthermore, the treatment significantly reduced urinary UA excretion, indicating normalization of the hyperuricemic state.

### 2.3. Combination Therapy with Empagliflozin/Metformin Attenuates Structural and Functional Cardiac Alterations in Cardio–Kidney–Metabolic Syndrome

Given that metabolic disorders, hypertension, and hyperuricemia are risk factors for heart failure, echocardiography analysis was performed to evaluate cardiac structure and integrity. The CKMS group exhibited a significant increase in both PWT and IVS compared to the control group (Figure 4A,B, *p* < 0.05 vs. control group). These alterations led to a significant elevation in relative wall thickness, indicative of concentric cardiac remodeling (Figure 4D *p* < 0.05 vs. control group). Notably, E+M co-therapy effectively reversed these changes, restoring PWT, IVS, and relative wall thickness to values close to those of the control group (Figure 4, *p* < 0.05 vs. CKMS group).

Taken together, these data show that, in the experimental model, cardiac structural and functional alterations associated with the CKMS were markedly improved by the combination of empagliflozin + metformin.

To further explore the underlying mechanisms, tissue concentrations of vasoactive substances Ang II and NO and total antioxidant capacity (TAC) were measured in cardiac homogenates.

The CKMS group exhibited significantly increased Ang II and NO concentrations compared with the control group (Figure 4E,F, *p* < 0.05 vs. control group), together with a significant reduction in cardiac TAC (Figure 4G, *p* < 0.05 vs. control group), indicating an altered vasoactive and redox profile. Treatment with E+M significantly reduced Ang II and NO levels compared with the untreated CKMS group (Figure 4E,F, *p* < 0.05 vs. CKMS group). However, cardiac Ang II levels remained significantly higher than those observed in the control group (Figure 4E). In contrast, E+M therapy significantly restored levels compared with the CKMS group (Figure 4G, *p* < 0.05 vs. CKMS group), reaching values close to those of the control group.

Overall, these results demonstrate that the empagliflozin+metformin combination confers cardioprotection at both structural and functional levels, likely through the modulation of vasoactive mediators and enhancement of antioxidant capacity.

### 2.4. Combination Therapy Attenuates Renal Dysfunction in Cardio–Kidney–Metabolic Syndrome

#### 2.4.1. Effects of Pharmacological Treatment on Renal Function

Evaluation of renal function markers in the CKMS group revealed marked increases in plasma creatinine, BUN, proteinuria, and diuresis (Figure 5A–D, *p* < 0.05 vs. control group). These findings, observed after 60 days of total follow-up, are consistent with the established biochemical and functional alterations associated with CKMS.

Following 30 days of treatment, the E+M combination significantly improved these renal markers, achieving values comparable to the control group (Figure 5A–D, *p* < 0.05 vs. CKMS group). To confirm the pharmacodynamic effect of empagliflozin on glucose reabsorption blockade, urinary glucose excretion was evaluated. As expected, the E+M-treated group showed a marked increase in glucosuria (Figure 5E, *p* < 0.05 vs. CKMS group), whereas no detectable urinary glucose was observed in either the control or untreated CKMS groups. These results underscore the therapeutic potential of empagliflozin/metformin combination in mitigating the renal impairment associated with CKMS.

#### 2.4.2. Effects of Pharmacological Treatment on Renal Anatomy and Histology

Because some cardiovascular risk factors such as hypertension and hyperuricemia impair renal function and may subsequently contribute to cardiac dysfunction, we further explored the effects of co-therapy on the kidneys. The CKMS group exhibited increased plasma creatinine and UA levels, suggesting altered glomerular function and possibly structure damage. In addition, it has been reported that in metabolic syndrome, obesity and hyperinsulinemia, through increased synthesis of growth factors such as IGF-1, promote glomerular hypertrophy [29,30]. Therefore, we calculated an index as an indicator of hypertrophy, analyzed glomerular size using H&E-stained sections, and detected IGF-1 protein expression, which was assessed by immunohistochemistry.

Kidney weight was recorded and used to calculate the hypertrophy index. The CKMS group presented a significant higher hypertrophy index compared with the control group, suggesting renal hypertrophy, which was effectively attenuated by empagliflozin/metformin co-therapy (Figure 6A, *p* < 0.05 vs. CKMS group).

Consistent with the statement that obesity and MetS promote glomerular growth through hyperinsulinemia and growth factors such as IGF-1 [29,30], morphometric and immunohistochemical analyses were performed. The CKMS group displayed a marked increase in glomerular size, characterized by glomerular hypercellularity and hypertrophy, in contrast to the normal architecture observed in the control group (Figure 6B,C). E+M therapy effectively prevented this glomerular enlargement, although values did not completely return to control levels (Figure 6B,C, *p* < 0.05 vs. CKMS group).

To further characterize the glomerular population, kernel density estimation (KDE) analysis was performed. The analysis revealed a rightward shift in the distribution of the CKMS group, moving from a healthy unimodal peak at 60 m in the control group to a broader distribution peaking at a 120–130 μm range, with some glomeruli reaching 200 μm (Figure 6D). This shift indicates diffuse glomerular hypertrophy likely driven by chronic hyperfiltration. Interestingly, the E+M treated group exhibited a bimodal distribution, with peaks at ranges of approximately 45–75 μm and 110–135 μm. This bimodal pattern reflects partial glomerular remodeling, in which a substantial subpopulation of nephrons underwent a reversal of the hypertrophic process. These structural improvements were further supported by IGF-1 expression analysis. The CKMS group showed intense IGF-1 immunostaining in numerous proximal convoluted tubules and mesangial cells (Figure 6E). In contrast, the E+M-treated group exhibited fewer IGF-1 positive cells and a complete absence of mesangial staining (Figure 6F). These results highlight the efficacy of E+M therapy in attenuating the morphological deterioration caused by CKMS and protecting the glomerular filtration barrier.

### 2.5. Renal Oxidative Stress and Antioxidant Status in the Kidneys

#### 2.5.1. Impact of Empagliflozin/Metformin Therapy on Oxidative Pathogenic Mechanisms

In CKMS cases, the convergence of hyperglycemia, IR, dyslipidemia, hypertension, and hyperuricemia triggers metabolic pathways that promote renal oxidative damage and contribute to endothelial dysfunction and vasoconstriction. Therefore, we first evaluated renal vasoactive balance and observed a significant increase in renal Ang II levels accompanied by a decrease in NO concentrations in the CKMS group (Figure 7A,B, *p* < 0.05 vs. control group), suggesting a predominance of renal vasoconstriction mechanisms.

Subsequently, mitochondrial integrity was assessed by measuring the activities of respiratory complexes I and II. The CKMS group exhibited a significant loss of activity of both complexes (Figure 7C,D, *p* < 0.05 vs. control group), indicating severe mitochondrial dysfunction. In contrast, treatment with the E+M combination significantly mitigated this impairment, restoring complex activities to values close to those of the control group (Figure 7C,D, *p* < 0.05 vs. CKMS group). Furthermore, lipid peroxidation, assessed by malondialdehyde (MDA) levels, was markedly elevated in the renal cortex of the CKMS group but was effectively reduced following E+M treatment, reaching levels comparable to those of the control group (Figure 7E, *p* < 0.05 vs. CKMS group).

#### 2.5.2. Restoration of the Nrf2-Mediated Antioxidant Defense

We delved deeper into the study of the responsible mechanisms for oxidative stress, and for this purpose, the activity of antioxidant enzymes GPx, GR, and SOD2, as well as the expression of Nrf2, were assessed as an indicator of endogenous antioxidant activity.

The CKMS group showed significant decreases in GPx, GR, and SOD2 activities compared with the control group (Figure 7F–H, *p* < 0.05 vs. control group), indicating a marked redox imbalance associated with CKMS. In contrast, treatment with the E+M combination attenuated the reduction in antioxidant enzyme activities compared with the untreated CKMS group (Figure 7F–H, *p* < 0.05 vs. CKMS group).

Thereafter, we purified nuclear extracts to analyze Nrf2 protein expression, the key regulator of the endogenous cellular antioxidant defense system. The control group exhibited highly nuclear Nrf2 protein expression, demonstrating appropriate physiological activation of endogenous antioxidant pathways. In contrast, the CKMS group presented a significant decrease in nuclear Nrf2 protein expression (Figure 8A, *p* < 0.05 vs. control group), which correlates with impaired antioxidant defense under conditions of oxidative stress induced by the dysfunctional metabolic environment. Importantly, combination treatment significantly restored nuclear Nrf2 expression (Figure 8A, *p* < 0.05 vs. CKMS group), reaching levels comparable to those observed in the control group. These results demonstrated that combination therapy can effectively restore the Nrf2-mediated endogenous antioxidant system, supporting its potential as a therapeutic strategy to mitigate oxidative damage under CKMS conditions.

#### 2.5.3. Polyol Pathway Activation

IR and glycolytic disturbances associated with CKMS, together with elevated UA levels in plasma and kidney tissue, contribute to increased oxidative stress. In the CKMS group, IR induces transient episodes of hyperglycemia, as observed during the OGTT, which stimulate glucose reabsorption at the level of the proximal renal tubules. Hyperglycemia is known to stimulate AR and SDH, the key enzymes of the polyol pathway. Overactivation of AR consumes NADPH, a critical cofactor required for regenerating reduced glutathione (GSH), whereas SDH activity increases the NADH/NAD+ ratio, thereby further promoting oxidative stress. We assessed AR and SDH protein expression as contributors to oxidative stress in CKMS individuals. The CKMS group showed increased renal expression of both AR and SDH. In contrast, combined treatment with E+M significantly downregulated the expression of these enzymes (Figure 8A, *p* < 0.05 vs. CKMS), suggesting that combination therapy improves cellular redox status, at least in part, through modulating the polyol pathway.

#### 2.5.4. Effect of Combined Empagliflozin/Metformin Therapy on Renal Lipogenic Factors

Metabolic dysfunction is characterized by profound alterations in carbohydrate and lipid metabolism, largely mediated by the transcription factors ChREBP and SREBP1. Our results revealed a significant upregulation of both ChREBP and SREBP1 in the renal tissue of the CKMS group (Figure 8B, *p* < 0.05 vs. control group), indicating a pathological activation of de novo lipogenic pathways in the kidneys. The therapy significantly reduced ChREBP expression compared to the untreated CKMS group (Figure 8B, *p* < 0.05 vs. CKMS group), although expression levels remained slightly above those of the control group. Conversely, despite a downward trend, E+M therapy did not significantly decrease SREBP1 expression, which remained elevated relative to the controls (Figure 8B, *p* < 0.05 vs. CKMS group).

These findings suggest that while the combination therapy effectively targets carbo-hydrate-mediated lipogenic signaling (ChREBP), persistent SREBP1 expression may reflect ongoing lipid accumulation within the renal parenchyma. Therefore, a longer treatment duration might be necessary to fully reverse the lipotoxicity associated with established CKMS.

#### 2.5.5. Effect of Combined Empagliflozin/Metformin Therapy on Inflammation in the Kidneys

The coexistence of metabolic, hemodynamic, and humoral alterations at both systemic and local levels activates inflammatory mechanisms, contributing to the progression of CKMS. We analyzed the expressions of IL-6, IL-10, TNFα, and NF-κB as indicators of renal inflammatory status. Our results demonstrated increased levels of IL-6, TNFα, and NF-κB in the kidney cortex of the CKMS group (Figure 9, *p* < 0.05 vs. control group), whereas IL-10 expression was decreased in the C KMS (Figure 9, *p* < 0.05 vs. control group). Combined E+M treatment reversed all inflammatory marker alterations observed in the CKMS group (Figure 9, *p* < 0.05 vs. CKMS).

Together, these data suggest a pro-inflammatory cellular environment that is further exacerbated by the impairment of anti-inflammatory mechanisms, which further exacerbates cardio–renal complications induced by metabolic and hemodynamic alterations.

## 3. Discussion

The present study characterizes the therapeutic efficacy of combined E+M treatment in a novel experimental model of CKMS. Our results demonstrate that 30 days of HFD supplemented with SSB successfully established a complex pathological phenotype characterized by obesity, glucose intolerance, hyperuricemia, systemic hypertension, and significant structural and functional impairments of both the heart and kidneys.

The most relevant finding of this research is that the E+M combination effectively reversed metabolic derangements and attenuated organ damage. Specifically, the therapy restored cardiorenal redox balance through a synergistic mechanism involving reactivation of the Nrf2 pathway, the suppression of key polyol pathway enzymes, and the restoration of mitochondrial bioenergetics. Furthermore, the use of advanced morphometric tools, such as KDE analysis, provided unprecedented evidence of the partial reversibility of glomerular hypertrophy under this pharmacological regimen. In addition, the empagliflozin+metformin combination decreased proinflammatory cytokines and restored IL-10 expression, likely mediated through the modulation of NF-κB expression. Collectively, these data suggest that the E+M combination targets key pathogenic intersections of CKMS, offering a robust therapeutic strategy to mitigate the progression of cardiorenal complications.

### 3.1. The Synergistic Effect of E+M on Cardiorenal Alterations Associated with CKMS

The empagliflozin/metformin combination successfully attenuated the full spectrum of metabolic derangements associated with CKMS, including dyslipidemia, impaired fasting blood glucose, glucose intolerance, hyperuricemia, hypertension, and cardiac and renal dysfunction. In clinical settings involving obese patients with type 1 diabetes mellitus (T1DM), the E+M combination has been shown to improve glycemic control, reduce HbA1c, and lower body weight, alongside reducing serum urea, creatinine, UA, and proteinuria [31,32,33,34]. Our experimental findings are consistent with these clinical outcomes, suggesting that dual therapy may target central pathogenic mechanisms underlying CKMS.

A key finding of our study was the restoration of UA homeostasis. Hyperuricemia is a dynamic mediator of cardiorenal injury, promoting hypertension and vascular damage through the activation of RAAS, NOX, increased ROS production, and the suppression of NO bioavailability, accompanied by inflammatory pathways mediated by the activation of NF-κB, and the promotion of cellular proliferation [14,35,36]. We observed that E+M treatment restored plasma UA levels to values comparable to the control group, in agreement with previous reports by Vuraloglu et al. and Bogoviku et al. [37,38], as well as a meta-analysis confirming the UA-lowering efficacy of SGLT2i [39]. This effect may be associated with the modulation of renal transporters, including organic cation transporters (OCTs) and multidrug and toxin extrusion protein 1 (MATE1), which are commonly dysregulated under hyperuricemic conditions [40].

### 3.2. Hemodynamic Stability and Cardiac Protection

The significant decrease in SBP observed following combined E+M therapy is consistent with the well-stablished natriuretic and osmotic diuretic effects of empagliflozin [41,42,43]. Furthermore, metformin contributes to this antihypertensive effect by improving endothelium-dependent relaxation (EDR) and upregulating NO and endothelium-derived hyperpolarizing factor (EDHF) in the aorta [44,45].

At the structural level, E+M therapy reversed LVH and improved diastolic function, as evidenced by the restoration of the PWT, LVDD, and the relative wall thickness. These results align with clinical trials where empagliflozin (10 mg/day) improved diastolic function, reduced left ventricular end-systolic dimension (LVESD), and elevated left ventricular end-diastolic pressure [46], even in patients with heart failure and preserved ejection fraction (HFpEF) [46,47,48]. Our data suggest that, through the reduction in hemodynamic overload and systemic inflammation, E+M therapy provides a robust cardioprotective effect [49]. Moreover, the combination decreased Ang II levels while restoring NO concentrations and TAC, suggesting improved cardiac vasodilation at both local and systemic levels, as reflected by a decrease in SBP. Of note, while LVDD exhibited a modest reduction at the early 30-day checkpoint (Figure 1C) due to acute concentric constraint, prolonged chronic exposure over 60 days led to compensatory cavity accommodation (Figure 4C), even as pathological wall thickening persisted.

The rationale for combining empagliflozin and metformin extends beyond traditional glycemic control. In addition to its primary renal action as an SGLT2 inhibitor inducing glucosuria and natriuresis, empagliflozin exerts direct cardioprotective effects. Emerging evidence demonstrates that empagliflozin prevents heart failure and reverses adverse cardiac remodeling—even in non-hyperglycemic or non-ischemic settings—partly through the inhibition of the myocardial sodium–hydrogen exchanger 1 (NHE1) pathway [50] and enhancement of cardioprotective autophagy [51]. These actions prevent intracellular calcium overload, improve mitochondrial bioenergetics, and directly mitigate myocardial oxidative stress and hypertrophy [52]. Simultaneously, metformin activates AMP-activated protein kinase (AMPK) pathways, modulates mitochondrial dynamics, and promotes cellular protection against ischemia-reperfusion injury and multi-organ damage [53]. The concurrent administration of both agents thus offers a robust multi-targeted approach addressing the complex multisystemic pathophysiology of cardio–kidney–metabolic syndrome.

### 3.3. Glomerular Remodeling and the IGF-1 Axis

In the CKMS group, we observed a rightward shift in glomerular diameter distribution (up to 200 μm), representing a compensatory yet ultimately maladaptive response to glomerular hyperfiltration [54,55]. The E+M combination effectively normalized glomerular size, presenting a bimodal distribution (KDE analysis) that suggests a subpopulation of nephrons underwent full structural recovery, with a glomerular diameter of 60 μm.

Glomerular hypertrophic remodeling is intrinsically linked to the modulation of growth factors. Hyperinsulinemia, IR, and obesity in MetS individuals stimulate IGF-1 synthesis, a potent trigger for mesangial cell proliferation and extracellular matrix accumulation, and it has been suggested that these pathological responses trigger renal hypertrophy [29,30,56,57]. In this study, E+M therapy significantly reduced IGF-1 overexpression.

The diet-induced model is well-established for inducing an early-to-intermediate phase of metabolic glomerulopathy and cardiorenal syndrome. As demonstrated by our functional data (hypertension and proteinuria), this phenotype is associated with impaired renal hemodynamics characterized by hyperfiltration and glomerular hypertrophy and significant albuminuria. While our histological analysis (presented in Figure 6) focused on key structural alterations—such as general glomerular architecture, cellularity distribution, and tubular appearance—we acknowledge that formal quantitative scoring for advanced glomerulosclerosis and tubular damage was not assessed. However, we analyzed the expression of molecular markers of glomerular (synaptopodin) and tubular injury [kidney molecular injury-1 (Kim-1) and claudin-2): Supplementary File]. The data showed increased synaptopodin and Kim-1 expression in the kidney cortex of the CKMS group, whereas claudin-2, a tight junction protein in the proximal tubules, was decreased.

Because podocytes have minimal division capacity, cytoskeletal stress and structural disruption lead directly to proteinuria and progressive kidney disease [58]. Under conditions of metabolic stress and hemodynamic overload, alterations in synaptopodin expression and its spatial reorganization reflect podocyte cytoskeletal stress and filtration barrier adaptation [59]. Our data showed increased renal synaptopodin expression, which may reflect a cytoskeletal response to the hemodynamic and metabolic stress associated with CKMS. Notably, E+M treatment attenuated this increase toward basal levels, concomitant with reduced proteinuria and normalization of glomerular dimensions. This effect may be related to the renoprotective actions of SGLT2 inhibition, which restores tubuloglomerular feedback, reduces intraglomerular pressure, even blood pressure (the most important secondary cause of microvascular damage and kidney failure) [60], but also to metformin´s ability to activate AMPK and attenuate oxidative stress in podocytes [61]. Collectively, these findings suggest that E+M may reduce podocyte stress, preserve glomerular barrier integrity, and attenuate tubular damage in CKMS.

On the other hand, metformin has been reported to improve peripheral insulin sensitivity through AMPK activation, thereby reducing hyperinsulinemia levels and systemic IGF-1 production [62]. Likewise, empagliflozin has been shown to mitigate extracellular matrix deposition by lowering renal IGF-1 levels [63]. This dual action likely prevents the progression of focal glomerulosclerosis and podocyte injury [31,64,65,66].

### 3.4. Mitochondrial Bioenergetics and Nrf2-Mediated Antioxidant Defense

The renoprotective effects observed in this study are closely linked to the restoration of redox homeostasis. The CKMS group exhibited a marked reduction in GPx, GR, and SOD activities, indicating a failure of the endogenous antioxidant defense system [67]. This imbalance was accompanied by mitochondrial dysfunction, particularly impaired activity of complexes I and II, compromising electron transport efficiency, promoting electron leakage, and enhancing ROS generation, ultimately leading to a bioenergetic deficit [68,69]. In addition, reduced complex II activity may favor succinate accumulation, a metabolite associated with altered mitochondrial signaling and lipid deposition under metabolic stress conditions [70]. Furthermore, evaluation of AR and SDH protein expression provided insight into the activation of the polyol pathway as a source of oxidative stress during transient hyperglycemic conditions. Together, these pathways reflect key mechanisms linking metabolic stress, redox imbalance, and structural kidney damage.

Treatment with empagliflozin plus metformin effectively improved mitochondrial bioenergetics, as evidenced by the restoration of complex I and II activities, reduction in MDA levels, and an increase in TAC. These effects were accompanied by increased Nrf2 protein expression in nuclei, supporting the involvement of Nrf2-mediated antioxidant response and suggesting a role in the recovery of cellular redox balance.

Additionally, suppression of the polyol pathway by E+M therapy represents a critical metabolic adaptation. By inhibiting AR, the therapy preserves NADPH availability, an essential cofactor required by GR for reduced glutathione (GSH) regeneration [9,71,72]. Together, these mechanisms may further support antioxidant capacity and mitigate oxidative stress.

These findings align with clinical studies evaluating combined empagliflozin and metformin therapy, which showed increased levels of the endogenous antioxidants [total antioxidant status (TAS), SOD, and GPx], together with reduced levels of pro-oxidant markers such as (AOPP and isoprostanes), as well as inflammatory markers [C-reactive protein (CRP) and IL-6] [31].

### 3.5. Selective Inhibition of Lipogenic Factors

We observed a state of metabolic dysregulation in the CKMS group characterized by the overexpression of ChREBP and SREBP-1c, resulting in lipo-glucotoxicity-mediated oxidative stress [73,74]. Increased lipid flow and de novo lipogenesis (DNL) contributed to renal lipotoxicity. Notably, E+M co-therapy significantly reduced ChREBP expression. This effect is consistently described for both empagliflozin [75] and metformin [76] treatments by inducing AMPK activation, therefore reducing ChREBP nuclear translocation and transcriptional activity. ChREBP inhibition has been shown to prevent the expression of carbohydrate-dependent lipogenic genes such as fatty acid synthase (FASN), acetyl-coA carboxylase (ACC), L-type pyruvate kinase (L-PK), and steroyl-CoA desaturase (SCD) [76,77,78,79,80]. In addition, metformin can decrease ChREBP through its direct activator, the carbohydrate metabolite glucose-6-phosphate (G6P) [81].

In contrast, SREBP1 was not modified with the E+M combination. This suggests that while glucose activated lipogenesis (ChREBP) is rapidly sensitive to SGLT2 inhibition and metformin [75,76,77,78], chronic lipid accumulation mediated by SREBP1 may require a longer treatment window to fully resolve. A protein regulated by SREBP1 is lipoprotein lipase (LPL), which plays a key role in lipid metabolism.

### 3.6. Inflammation in CKMS Progression

In the present study, the CKMS group exhibited a pronounced pro-inflammatory profile in the renal cortex, characterized by the marked overexpression of IL-6, TNF-α, and NF-κB, whereas the anti-inflammatory cytokine IL-10 was significantly downregulated.

The renal inflammatory cascade is directly triggered by metabolic and hemodynamic overload [82]. In agreement with our biochemical findings, persistent hyperglycemia, elevated UA concentrations and renal levels of Ang II, as well as impaired activity of endogenous antioxidant systems activate the NF-κB signaling pathway [14,35,36]. NF-κB activation promotes the transcription of the pro-inflammatory cytokines IL-6 and TNF-α, thereby enhancing leukocyte recruitment, exacerbating endoplasmic reticulum stress, and promoting extracellular matrix accumulation [36,83,84,85]. This inflammatory state contributes to localized renal injury by promoting endothelial dysfunction, increasing vascular permeability, and disrupting the integrity of the glomerular filtration barrier. Elevated levels of TNF-α and IL-6 have been shown to induce podocyte injury and apoptosis, stimulate mesangial cell proliferation and extracellular matrix deposition, and accelerate the development of glomerulosclerosis [86,87]. Consequently, this inflammatory microenvironment represents a key mechanistic link between systemic metabolic stress and the structural remodeling and progressive functional decline observed in the kidneys during CKMS progression [87,88].

Given the central role of NF-κB in sustaining this inflammatory network, attenuation of its activation represents an important therapeutic target in CKMS. Consistent with this concept, treatment with the E+M combination markedly attenuated the intrarenal inflammatory response, significantly reducing NF-κB, TNF-α, and IL-6 protein expression while restoring IL-10 levels to values comparable to the control group (Figure 9). Mechanistically, these anti-inflammatory effects are consistent with the restoration of renal antioxidant defenses observed in the present study, including the recovery of GPx, SOD, and GR activities. Because oxidative stress is a major upstream activator of NF-κB, these findings suggest that E+M therapy disrupts the ROS-NF-κB inflammatory axis, thereby limiting pro-inflammatory cytokine production and contributing to the preservation of renal homeostasis [89,90]. Furthermore, restoration of IL-10 likely reinforced this protective response by counteracting NF-κB-dependent signaling, limiting inflammatory cell recruitment, and reducing extracellular matrix deposition, thereby creating a more favorable intrarenal microenvironment [91]. Collectively, these findings suggest that coordinated attenuation of oxidative stress and inflammatory signaling contributes to the renoprotective effects of E+M therapy, preserving glomerular integrity and renal function during CKMS progression.

In the present study, we evaluated combination therapy exclusively, based on previous findings demonstrating that the most pronounced hepatoprotective effects were achieved with the combined treatment compared to the respective monotherapies [92]. In addition, other studies have reported that co-administration of SGLT2i (empagliflozin, dapagliflozin) and metformin yields superior renal and biochemical levels than the treatments individually, specifically regarding inflammation, oxidative stress, apoptosis, activation of autophagy by modulating the AMPK/mTOR/SIRT1 axis, and the improvement of renal function markers in diabetes [33,93]. Furthermore, histological assessments revealed that renal tissues in combination-treated groups maintained a near-normal architecture, effectively preventing diabetes-induced glomerular and tubular damage [33]. Notably, while that study evaluated the combination as a preventive strategy, our work assessed its efficacy in a therapeutic framework. Interestingly, the pleiotropic benefits of SGLT2i/metformin combination therapy extend to other tissues, including the pancreas, liver, and heart [28,33,92,93]. This finding supports the notion that combination therapy provides greater efficacy over monotherapy in conditions where multiple target organs undergo concomitant metabolic and hemodynamic insult, such as in cardiorenal metabolic diseases [28,94].

### 3.7. Limitations and Caveats

This study was conducted exclusively on male Wistar rats to control for the metabolic and hemodynamic fluctuations associated with the female estrous cycle. Given that cardio–kidney–metabolic syndrome exhibits notable sex-specific differences in pathophysiology and therapeutic responses, future studies are warranted to evaluate the efficacy of the empagliflozin and metformin combination in female animal models. Also, future studies should incorporate direct measurements of ROS and more robust, validated oxidative stress markers, such as *F*_2_-isoprostanes, to more precisely evaluate cellular redox status. However, in our study, we evaluated oxidative stress through multiple complementary approaches, including the assessment of TAC; mitochondrial complex I and II activities; GPx, GR, and SOD2 activities; and nuclear Nrf2 expression. The observed alterations in these parameters are consistent with impaired endogenous antioxidant defences and, consequently, elevated oxidative stress.

Our biochemical analyses quantified stable nitric oxide metabolites (nitrites/nitrates) rather than direct real-time levels of the short-lived nitric oxide radical. Future studies should incorporate direct measurements of nitric oxide via electron paramagnetic resonance (EPR) spectroscopy, together with functional assessments of NO-mediated vasorelaxation, to gain deeper mechanistic insights into vascular endothelial dynamics.

A key limitation of this study is its short 30-day treatment period, which restricts insights into long-term remodeling processes, including fibrosis, and inflammation. Nevertheless, this timeframe allowed the identification of the main molecular and functional mechanisms involved in early protective effects. Extended studies should evaluate prolonged treatment and further investigate fibrotic and inflammatory pathways, including phosphoinositide 3-kinase (PI3K)/serine/threonine-specific protein kinase (Akt) and RAAS signaling, to clarify chronic adaptative responses. Another limitation is that mitochondrial function was evaluated exclusively through complexes I and II, which represent the primary electrons’ entry points into the electron transport chain and are particularly sensitive to oxidative stress-induced dysfunction. However, other respiratory chain components (complexes III–V) were not assessed. In addition, mitochondrial activity was not normalized to citrate synthase activity, which limits the ability to distinguish between alterations in mitochondrial function and changes in mitochondrial mass.

Equally important, future studies should explore in greater depth the antihypertensive, antioxidant, profibrotic, and even inflammatory mechanisms of the heart, which we did not thoroughly explore, nor did we determine whether they were extensive in the endothelium, heart, or other organs. Therefore, additional studies exploring upstream signaling pathways—including AMPK activation, mitochondrial regulatory mechanisms, antihypertensive and antidyslipidemic mechanisms, as well as tissue-specific responses—will be essential to better define the mechanistic basis underlying the protective effects observed with this therapeutic combination. Furthermore, while structural and functional cardiac parameters were evaluated in vivo via high-resolution transthoracic echocardiography and tissue biochemistry, dedicated myocardial histology was not performed for this cohort due to tissue prioritization of biochemical and redox analyses. Finally, while this short-term dietary intervention reflects an early-to-intermediate phenotype, it provides critical insights into early functional and molecular rescue. Future long-term investigations with dedicated structural tissue collection will be essential to map chronic histological alterations.

The combination of empagliflozin and metformin holds promise as a therapeutic strategy for CKMS management due to its complementary metabolic effects; urate-lowering properties; vasodilative, antioxidant, and anti-inflammatory activities; and structure-protective mechanisms. Nevertheless, long-term preclinical and clinical studies are necessary to validate these findings and assess the efficacy of this dual therapy in reducing cardiorenal complications in patients with CKMS.

Despite these limitations, the combination of empagliflozin and metformin shows promising cardio–renal protective effects through complementary metabolic, antioxidant, anti-inflammatory, and structural mechanisms. However, long-term preclinical and clinical studies are required to confirm these findings and evaluate their impact on cardiorenal outcomes in CKMS individuals.

## 4. Materials and Methods

### 4.1. Experimental Design

Male Wistar rats in the weight range of 200–220 g were housed in polypropylene cages under controlled conditions, with a 12:12 h light/dark cycle and an ambient temperature maintained at 22 ± 2 °C. Animals were randomly assigned to two groups: a control group (C) (n = 6), which received Labdiet 5001 Rodent Diet^TM^ and water ad libitum, and a CKMS group (n = 12) on a Paigen-type (high-fat) diet supplemented with an 11% sweetened beverage (SSB) (3.8% glucose:7.2% fructose) administered ad libitum, as previously described [5].

After 30 days of dietary induction, establishment of the CKMS phenotype was verified by evaluating baseline metabolic, cardiovascular, and renal parameters (n = 6, control vs. n = 12 CKMS; reported in Table 1 and Figure 1). Following baseline validation, the 12 CKMS animals were randomized in a 1:1 ratio into two parallel groups (n = 6 each) for a 30-day treatment period (days 30 to 60): (1) the untreated CKMS group (n = 6), which continued the Paigen-type diet + SSB and received the vehicle (1% hydroxyethyl cellulose, HEC) by oral gavage; and (2) the combined treatment group (E+M, n = 6), which continued the diet + SSB and received empagliflozin plus metformin (12.5/850 mg/kg/day/30 days, respectively) (Jardianz Duo) suspended in 1% HEC by oral gavage. The control group (n = 6) maintained standard chow, plain drinking water, and vehicle gavage from days 1 to 60. Consequently, all physiological, hemodynamic, serum, urinary, and echocardiographic end-point evaluations at day 60 were conducted using n = 6 animals per group, as previously described [5]. Drug solutions and vehicles were prepared in 1% hydroxyethyl cellulose (HEC), and an equivalent volume of HEC was administered to both control and untreated CKMS groups.

#### Drugs

All the chemicals used in the present study were of the highest available purity and were purchased from Sigma-Aldrich Products (St. Louis, MO, USA).

### 4.2. Validation of Cardio–Kidney–Metabolic Syndrome Establishment

To confirm the establishment of CKMS, metabolic alterations were evaluated based on the presence of a ≥20% difference in three or more risk factors (as MetS occurred) after 30 days of diet-induced CKMS. The assessed parameters included body weight, systolic blood pressure (SBP), fasting glucose, oral glucose tolerance test (OGTT), triglycerides, total cholesterol (TC), high-density lipoprotein cholesterol (HDL-c), or free fatty acids (FFAs), compared with the control group (maintenance diet and plain water). Animals that did not meet these criteria were excluded from this study. Briefly, prior to initiating the therapeutic intervention (at day 30 of the experimental protocol), a comprehensive baseline characterization was performed across all experimental groups to validate the established cardio–kidney–metabolic syndrome (CKMS) phenotype. Following a 12-hour fasting period, blood samples (500 µL) were collected via the tail vein to determine baseline fasting blood glucose, lipid profile (triglycerides, total cholesterol, HDL-c, and free fatty acids), serum creatinine, blood urea nitrogen (BUN), and uric acid. Systolic blood pressure (SBP) was recorded non-invasively using a high-precision tail-cuff plethysmography system after a standardized acclimatization period. Twenty-four-hour urine collections were obtained using individual metabolic cages to quantify baseline proteinuria, urinary volume, and urinary uric acid excretion. Furthermore, baseline in vivo cardiac structural and functional parameters—including posterior wall thickness (PWT), interventricular septal thickness (IVS), left ventricular diastolic diameter (LVDD), and relative wall thickness—were evaluated using high-resolution transthoracic echocardiography prior to animal randomization and treatment allocation.

#### 4.2.1. Clinical Chemistry

Blood samples were obtained on the days 30 and 60 of the follow-up. After an overnight fast, 1.0 milliliter of blood was collected from the tail vein and placed into heparin tubes. Samples were then centrifuged at 3500× *g* for 15 min at 4 °C for plasma separation. Plasma was then separated, aliquoted, and stored at −70 °C until further analysis.

Plasma samples were used to measure glucose, creatinine, blood urea nitrogen (BUN), uric acid (UA), and lipid profile parameters. The kits used had the following catalog numbers: glucose (Glucose-LQ, REF41012), triglycerides (Triglycerides LQ, REF: 41030), total cholesterol (Cholesterol LQ, REF: 41020), high-density lipoprotein cholesterol (HDLc-D, REF: 1001097), creatinine (Creatinine-J REF: 1001111), and uric acid (Uric acid-LQ REF: 41000) and were obtained from Spinreact (Girona, Spain). Free fatty acids (Free Fatty Acid Assay Kit, ab65341) were obtained from Abcam (Abcam Inc, Waltham, MA, USA) and BUN (EIABUN) from Thermofisher Scientific, Frederick, MD, USA. All analytes were measured according to the respective manufactures´ protocols using a multimode reader (BioTek Synergy H1, Santa Clara, CA, USA). Results are reported in the units specified by each manufacturer.

#### 4.2.2. Blood Pressure Recording

Systolic blood pressure (SBP) was assessed by the tail-cuff plethysmography method (IN125/R, ADI Instruments, Colorado Springs, CO, USA) in conscious, restrained rats at the following follow-up intervals: 30 and 60 days. Prior to SBP assessment, the rats were placed in an incubator at 40 °C for 5 min to facilitate tail vein dilatation. SBP values represent the mean of three consecutive measurements per rat and are reported in mmHg.

#### 4.2.3. Oral Glucose Tolerance Test (OGTT)

The oral glucose tolerance test (OGTT) was performed at days 30 and 60 after a 12 h fasting period. Blood samples were collected from the tail vein, and the basal glucose levels were measured using a FreeStyle Optium Neo Blood glucose and ketone monitoring system (Abbott). A glucose bolus (3 g/kg body weight) was then administered orally, and blood glucose concentrations were determined at different times: 0, 30, 60, 90, 120, and 150 min in all experimental groups. OGTT results are expressed as the area under the curve (AUC), calculated using the Trapezoidal Rule, which estimates cumulative glucose exposure by summing the areas of the trapezoids formed between consecutive time points.

#### 4.2.4. Cardiac Function Assessed by Echocardiography

Echocardiography analysis was performed on days 30 and 60. Rats were anesthetized with isoflurane (5% induction, 2.0% maintenance) and subjected to transthoracic echocardiographic using a SONOS 550 ultrasound system (Koninklijke Philips Electronics, Eindhoven, The Netherlands) with a 12 MHz transducer. The following echocardiographic approximations were obtained in the two-dimensional mode and M mode: parasternal long-axis approximation, parasternal short-axis approximation, and apical 4- and 5-chamber views. Left ventricular systolic function was assessed by calculating the ejection fraction (EF) and shortening fraction (FS).

#### 4.2.5. Assessment of Renal Function

Renal function was assessed using 24-h urine samples. On days 30 and 60, rats were placed in individual metabolic cages (Nalgene, Rochester, NY, USA) for 24 h with free access to food and water. The collected urine was centrifugated at 5000× *g* for 15 min. Afterward, the urine was divided into aliquots and stored at −70 °C until further analysis. Parameters measured included diuresis, urinary glucose excretion, creatinine, urea, uric acid, and proteinuria.

### 4.3. Obtention of Renal Tissue

After 30 days of drug treatment or 60 days from study initiation, all animals were anesthetized with sodium pentobarbital (65 mg/kg i.p.) and exsanguinated via the abdominal aorta. Kidneys were surgically removed, rinsed in cold 0.9% NaCl, weighed, and processed.

One kidney was sectioned sagittally; one half was fixed by immersion in 10% formaldehyde prepared in phosphate-buffered saline (PBS) for histologic analysis. The cortex and medulla of the remaining half were snap-frozen in liquid nitrogen and stored at −70 °C until further analysis.

Similarly, the heart was excised, rinsed in cold 0.9% NaCl, minced, snap-frozen, and stored at −70 °C until further analysis.

### 4.4. Assessment of Oxidative Stress

Oxidative stress was assessed using multiples indicators, including lipid peroxidation malondialdehyde (MDA), total antioxidant capacity (TAC), the activity of key antioxidant endogenous enzymes [superoxide dismutase (SOD), glutathione peroxidase (GPx), and glutathione reductase (GR)], mitochondrial complex I and II activities, and the nuclear Nrf2 expression in kidney tissue samples.

Additionally, TAC, nitric oxide (NO) concentrations, and the angiotensin II (Ang II) levels were determined in the heart and kidney homogenates according to the methods previously described [95].

#### 4.4.1. Measurement of Lipid Oxidation in Kidney Tissue

Malondialdehyde (MDA) concentrations were determined as an indirect index of lipid peroxidation. Samples were deproteinized with cold methanol (1:1 ratio), vortexed for 1 min, and then 0.1 M sodium hydroxide (NaOH) and ammonium acetate were added before direct analysis. MDA levels were quantified by high-performance liquid chromatography (HPLC) using a µBondapak C18 column (10 µm, 125% Å, 3.9 × 150 mm) at a flow rate of 0.4 mL/min for 10 min at a wavelength of 200 nm. A standard calibration curve (0 to 50 pmol/mL) was generated using MDA standards processed under identical conditions. MDA concentrations were calculated by interpolation with the standard curve [96].

#### 4.4.2. Antioxidant Enzyme Activities in Kidney Tissue

Antioxidant enzyme activities were measured according to the method previously described [5]. GR activity was evaluated by measuring the disappearance of nicotinamide adenine dinucleotide phosphate (NADPH) at 340 nm. GPx activity was determined by measuring the disappearance of NADPH at 340 nm in a coupled reaction with GR. GR and GPx unit (U) activity was defined as the amount of enzyme required to oxidize 1 micromol NADPH/min, and the results are expressed as U/mg total protein. SOD activity was determined spectrophotometrically at 560 nm using nitroblue tetrazolium (NBT) as the indicator reagent; the amount of protein that inhibited 50% of NBT reduction was defined as one unit of SOD activity. Results are expressed as U/mg protein of total protein. The changes in absorbance were read with a multimode reader (BioTek Synergy H1, Santa Clara, CA, USA).

#### 4.4.3. Mitochondrial Complex Activities in the Renal Cortex

After sacrifice, kidneys were excised and placed in ice-cold isolation buffer (225 mM D-mannitol, 75 mM sucrose, 1 mM EDTA, 5 mM HEPES, 0.1% BSA, pH 7.4) at 4 °C and then finely minced. Mitochondria were isolated using a Potter–Elvehjem glass homogenizer fitted with a Teflon plunger, followed by differential centrifugation through Percoll gradients. The final mitochondrial pellet was resuspended in 80 μL of a BSA-free isolation buffer, and mitochondrial protein concentration was determined using the Bradford assay. Mitochondrial complex activities were assessed as previously described [5]. Complex I activity was assessed by monitoring the oxidation of NADH coupled with the reduction of deubiquinone, using rotenone as a specific inhibitor, while complex II activity was evaluated through a coupled succinate deubiquinone oxide-reduction reaction in the presence of maleic acid as the inhibitor. In both assays, the changes in absorbance were read using a multimode reader (Sinergy H1; Biotek-Agilent, Santa Clara, CA, USA) [5]. The activity of mitochondrial complexes I and II was evaluated as representative markers of electron transport chain function, given their critical role in NADH and FADH_2_ oxidation and their high susceptibility to oxidative damage.

### 4.5. Histological Analysis

#### 4.5.1. Analysis of Glomerulopathy

Paraffin-embedded kidney tissues from a representative subset of animals (n = 3 biologically independent rats per group) were sectioned at 4 µm for histological and morphometric analyses. Sections were deparaffinized and rehydrated through successive passages in xylene and graded alcohols, followed by hematoxylin and eosin (H&E) staining. For morphometric evaluation, 20 images of maximal glomerular profiles, identified by the presence of the vascular and/or urinary pole, were captured from each animal at 400× magnification using a confocal microscope (totaling 60 glomeruli evaluated per experimental group). Glomerular tuft morphometry and mesangial cellular distribution were determined using Nikon NIS-Elements Basic Research image software (v. 3.0; Nikon Corporation, Tokyo, Japan). The two longest perpendicular diameters of each capillary tuft (excluding Bowman’s space) were measured to calculate the mean diameter per glomerulus and to construct the kernel density estimation (KDE) distribution plots.

#### 4.5.2. Immunohistochemistry

The same paraffin blocks with embedded kidney tissue were used for insulin-like growth factor 1 (IGF-1) detection by immunohistochemistry. Briefly, 4 µm thick tissue sections were placed on silanized slides. After deparaffinization and rehydration, antigen retrieval was performed using a citrate buffer solution (ImmunoDNA Retriever Citrate, Bio SB, Santa Barbara, CA, USA) by heat-induced epitope retrieval (HIER). Endogenous peroxidase activity was quenched by incubation with 0.3% hydrogen peroxide. Non-specific binding was blocked using Background Sniper (Biocare Medical, Pacheco, CA, USA). Kidney sections were incubated overnight at room temperature with specific IGF-1 rabbit antibodies diluted at 1:200 (IGF-1, GTX32670) (GeneTex, Irvine, CA, USA). Following primary antibody incubation, sections were treated with a mouse/rabbit polydetector DAB HRP system (Bio SB, Goleta, CA, USA). Immunoreactivity was revealed with diaminobenzidine (Merck) and counterstained with hematoxylin (Bio SB, Goleta, CA, USA).

### 4.6. Protein Expression Analysis

Kidney cortex samples (50 mg) were homogenized in a cold buffer containing 150 mM KCl, 20 mM Tris (pH 7.5), 1 mM EDTA (pH 8), and 1 mM DTT to assess the protein expression of aldose reductase (AR) and sorbitol dehydrogenase (SDH). To assess nuclear transcription factor expression (Nrf2, ChREBP and SREBP), we purified nuclear extracts according to the methods previously described [5]. Equal amounts of protein from total lysate of kidney cortex and nuclear extract were loaded on SDS-PAGE gels in a minigel system (Mini -PROTEAN II, Bio-Rad, Hercules, CA, USA) and transferred to PVDF membranes (0.4 μm pore size, Merck-Millipore). The membranes were blocked with 5% milk in TBS-Tween for 1 h and were incubated overnight at 4 °C with the following antibodies: Claudin 2 (SC-293233, Santa Cruz Biotechnology, Inc., Dallas, TX, USA), aldose reductase (AR) (GTX113381), sorbitol dehydrogenase (SDH) (GTX83588), nuclear factor erythroid 2-related factor 2 (Nrf2) (GTX103322), IL-6 (GTX110527), TNF-α (GTX110520), IL-10 (GTX632359), Kim-1 (GTX85067), synaptopodin (GTX85140), NF-κB p65 (GTX102090), GAPDH (GTX100118), carbohydrate-response element-binding protein (ChREBP) (GTX30677), and sterol regulatory element-binding protein (SREBP) (GTX79299). Proliferating cell nuclear antigen (PCNA) was used as the loading control (GTX100539) to Nrf2, ChREBP, and SREBP. The protein expressions of AR and SDH were corrected for β-actin (GTX637676, GeneTex Inc., Irvine, CA, USA). After incubation and washing steps, the secondary antibodies conjugated with horseradish peroxidase (HRP) were added. The proteins were detected by enhanced chemiluminescence (Clarity ECL Western Substrate, Bio-Rad, Hercules, CA, USA). Densitometry was performed with a ChemiDoc Imaging System (Bio-Rad, Hercules, CA, USA) and the data are expressed as arbitrary units.

### 4.7. Statistics

Results are presented as mean values ± SEM. The level for statistical significance was defined as *p* < 0.05. Analyses were carried out using GraphPad Prism Software version 9 (San Diego, CA). Differences between data groups were evaluated for significance using independent *t*-tests of data or one-way analysis of variance and Tukey’s post hoc tests for multiple comparisons.

## 5. Conclusions

Our findings demonstrate that the CKMS model effectively recapitulates a clinically relevant cardio–renal–metabolic state, characterized by profound metabolic disruption, hyperuricemia, hypertension, inflammation, and significant structural damage to the heart and kidneys. This pathological phenotype is driven by critical redox imbalance, a marked bioenergetic failure, and inflammatory mechanisms within renal tissue.

Therapeutic intervention with the empagliflozin/metformin combination proved highly effective in mitigating multi-organ damage. The protective effect was evidenced by the restoration of systemic metabolic homeostasis; the normalization of cardiovascular parameters, including the reversal of left ventricular hypertrophy; and a significant preservation of renal function and glomerular integrity.

At the mechanistic level, this dual therapy acted as a potent modulator of the renal microenvironment by: (1) attenuating oxidative stress, as evidenced by increased Nrf2 protein expression and enhanced endogenous antioxidant capacity, consistent with the involvement of the Nrf2-mediated antioxidant response; (2) improving mitochondrial function through increased activity of respiratory complexes I and II, thereby contributing to the restoration of cellular energy balance; (3) reprogramming metabolic flux via coordinated modulation of uricosuric, polyol, and lipogenic pathways; and (4) attenuating inflammatory cascades driven by the NF-kB signaling pathway.

Altogether, these results provide compelling evidence that the empagliflozin/metformin combination targets the central hubs of CKMS progression. Beyond its established metabolic benefits, this dual therapy offers a superior cardio–renal protective effect by preventing oxidative injury and preserving tissue architecture, positioning it as a highly promising therapeutic strategy for the integrated management of CKMS.

## Figures and Tables

**Figure 1:**
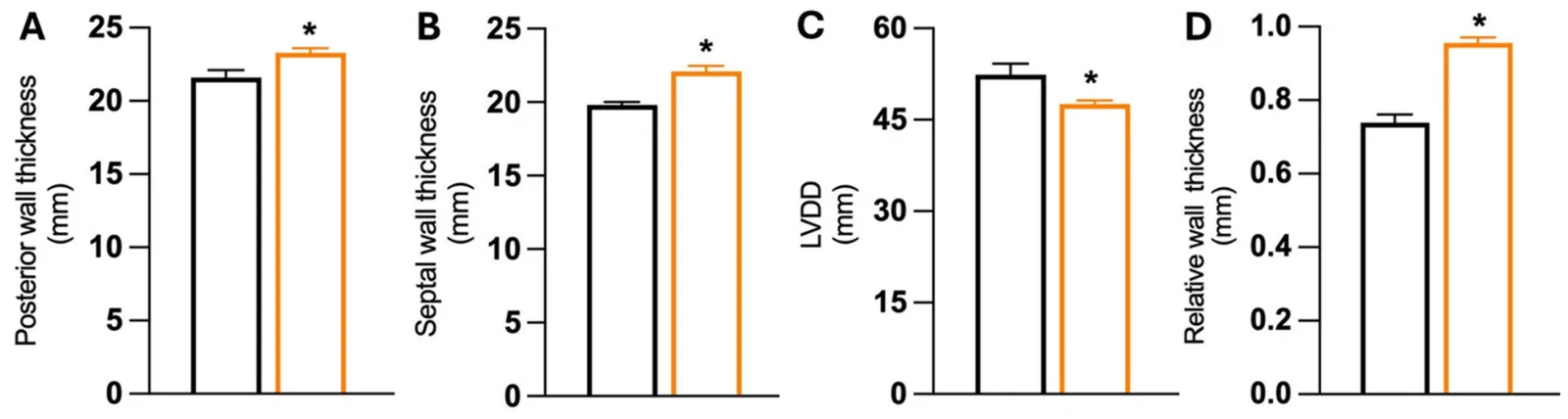
Assessment of cardiac function by echocardiography before initiating pharmacological treatment. (**A**) Posterior wall thickness (PWT), (**B**) interventricular septum/posterior wall (PP/PS) thickness ratio, (**C**) left ventricular diastolic diameter (LVDD), and (**D**) relative wall thickness. Open bars in black lines (□) represent the control group (Ctrl), and open bars in orange lines (□) correspond to the cardio–kidney–metabolic syndrome group (CKMS). Data are presented as mean ± SEM (n = 6). Statistical significance was established as * *p* < 0.05 vs. control group.

**Figure 2:**
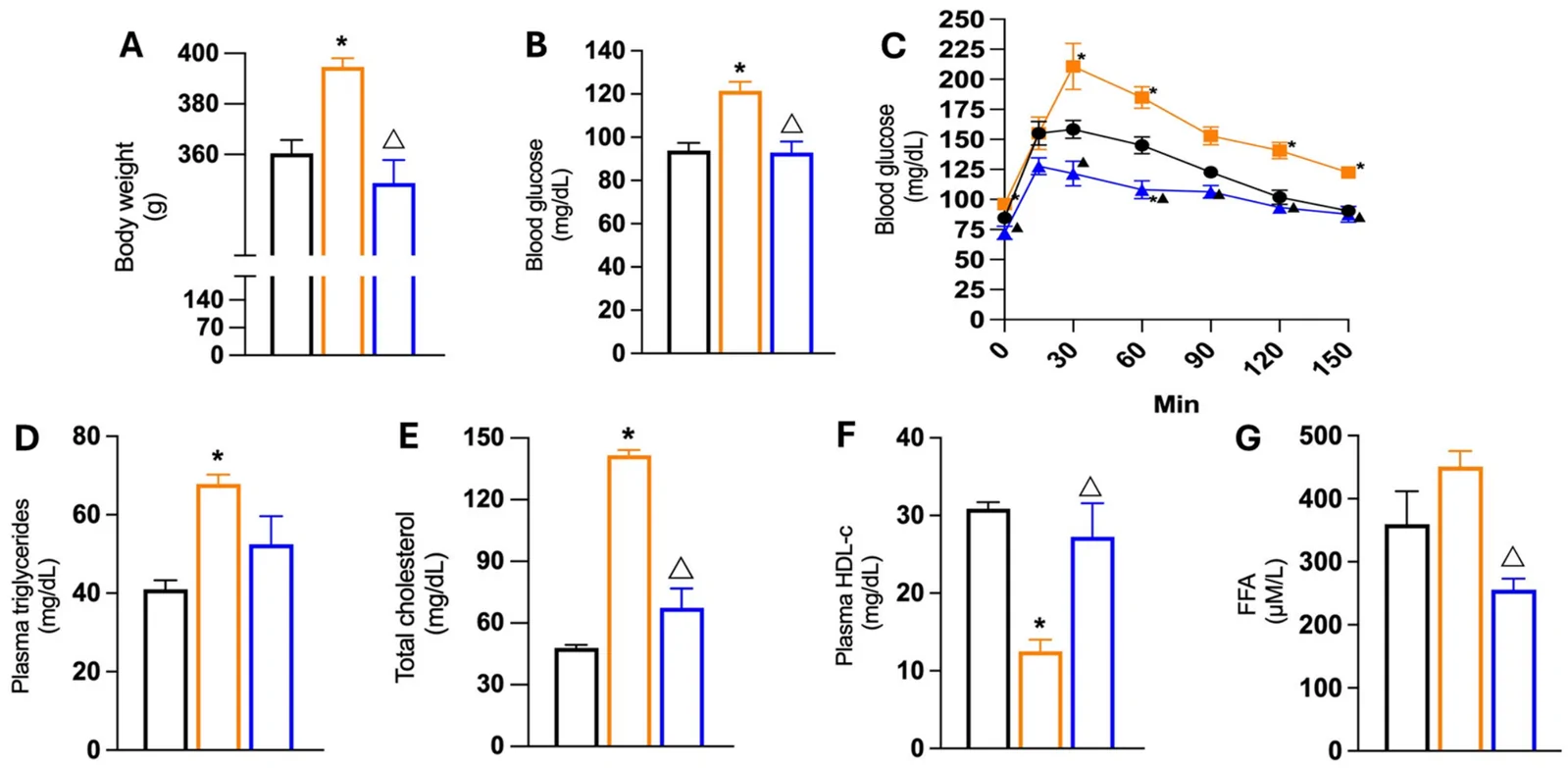
Effects on the biochemical profile after 30 days of treatment with the combination. (**A**) Body weight, (**B**) fasting blood glucose, (**C**) oral glucose tolerance test (OGTT), (**D**) plasma triglycerides, (**E**) total cholesterol, (**F**) plasma high-density lipoprotein cholesterol (HDL-c), and (**G**) free fatty acids (FFAs). Open bars in black lines (□) represent the control group (Ctrl), open bars in orange lines (□) correspond to the cardio–kidney–metabolic syndrome group (CKMS), and the bar in blue lines (□) represents the empagliflozin+metformin combination group (E+M). Data are expressed as mean ± SEM (n = 6) and analyzed by one-way ANOVA, followed by Tukey’s multiple comparison tests. * *p* < 0.05 versus control group; ^△^ *p* < 0.05 versus CKMS group.

**Figure 3:**
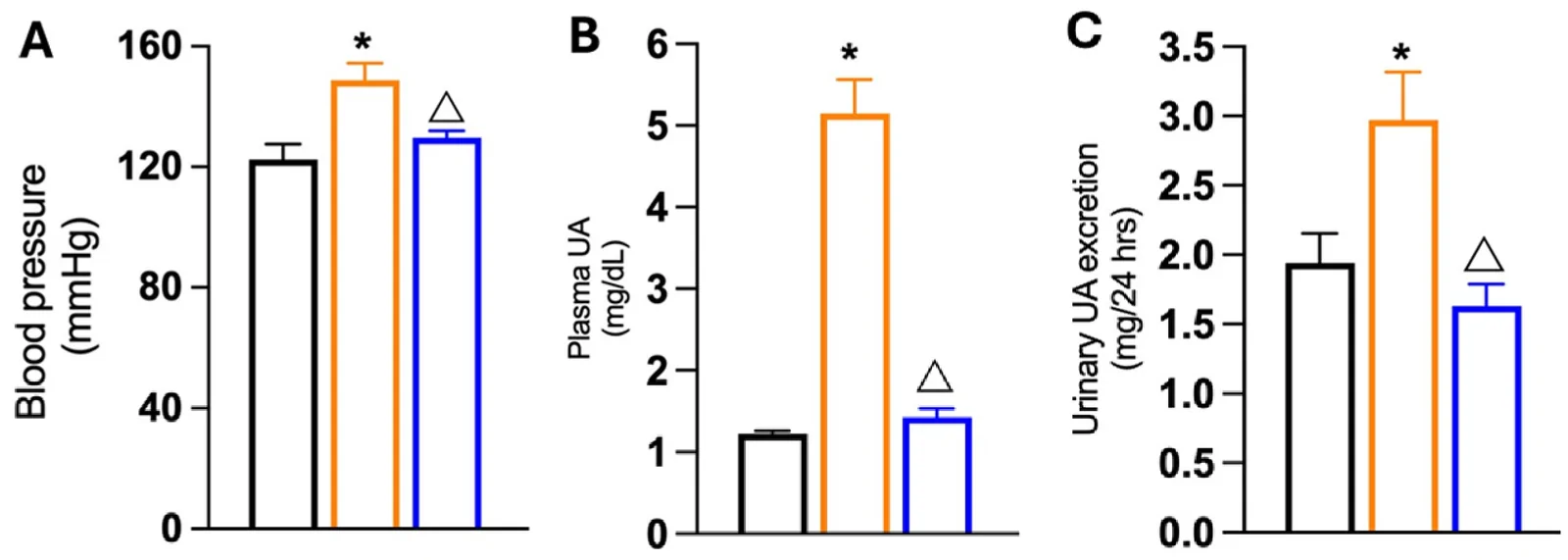
Effect of drug therapy on cardiovascular risk factors in CKMS patients. (**A**) Blood pressure, (**B**) plasma uric acid levels, and (**C**) urinary excretion of uric acid. Open bars in black lines (□) represent the control group (Ctrl), open bars in orange lines (□) correspond to the cardio–kidney–metabolic syndrome group (CKMS), and the bar in blue lines (□) represents the empagliflozin+metformin combination group (E+M). Data are expressed as mean ± SEM (n = 6) and analyzed by one-way ANOVA followed by Tukey’s multiple comparison test. * *p* < 0.05 vs. control group; ^△^ *p* < 0.05 vs. CKMS group.

**Figure 4:**
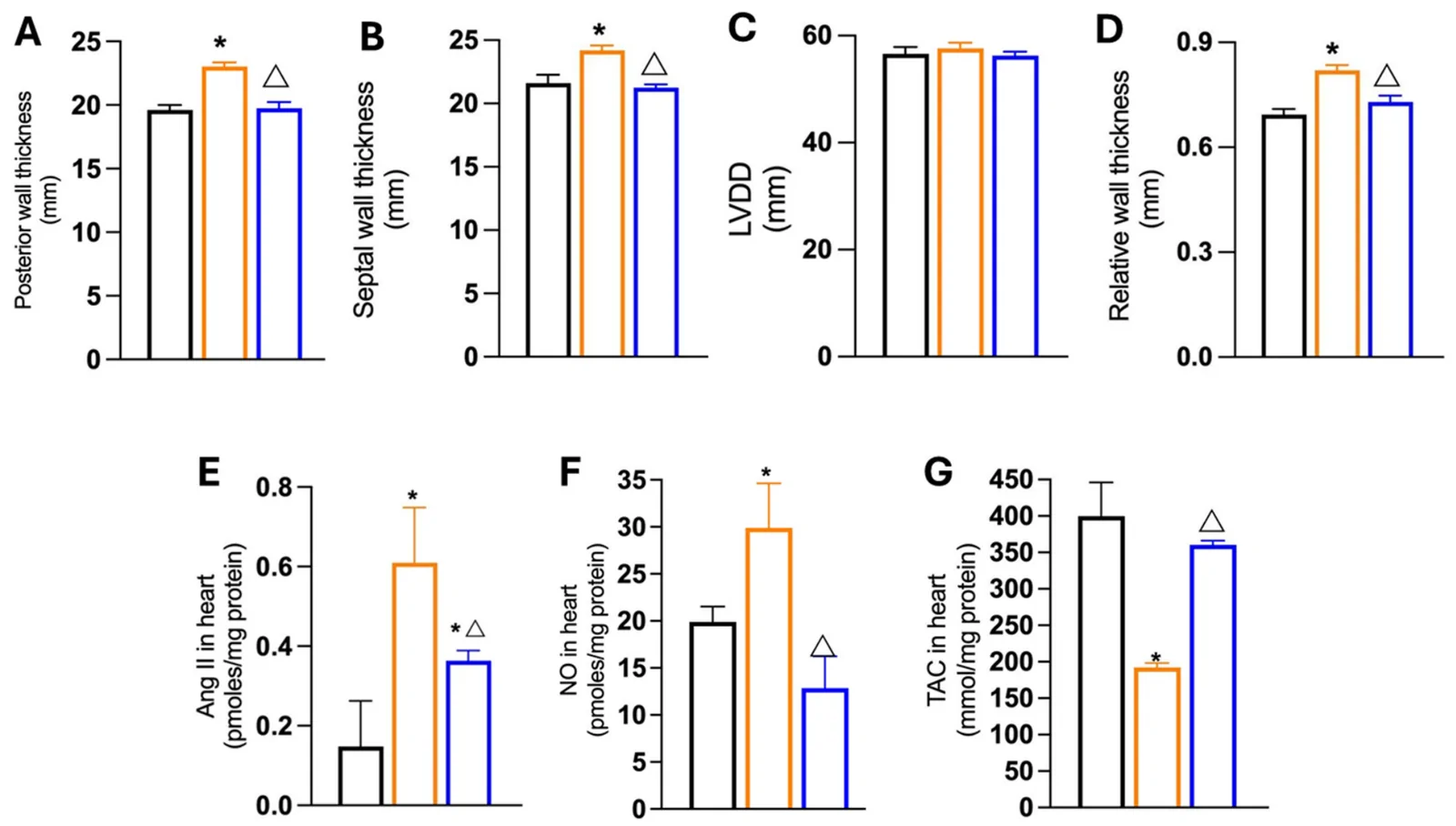
Assessment of cardiac function by echocardiography analyses and vasoactive profile. (**A**) Posterior wall thickness, (**B**) septal wall thickness, (**C**) left ventricular diastolic diameter (LVDD), (**D**) relative wall thickness, (**E**) angiotensin II concentration, (**F**) nitric oxide (NO) concentration, and (**G**) total antioxidant capacity (TAC). Open bars in black lines (□) represent the control group (Ctrl), open bars in orange lines (□) correspond to the cardio–kidney–metabolic syndrome group (CKMS), and the bar in blue lines (**□**) represents the empagliflozin+metformin combination group (E+M). Data are expressed as mean ± SEM (n = 6) and analyzed by one-way ANOVA followed by Tukey’s multiple comparison tests. * *p* < 0.05 vs. control group; ^△^ *p* < 0.05 versus CKMS group.

**Figure 5:**
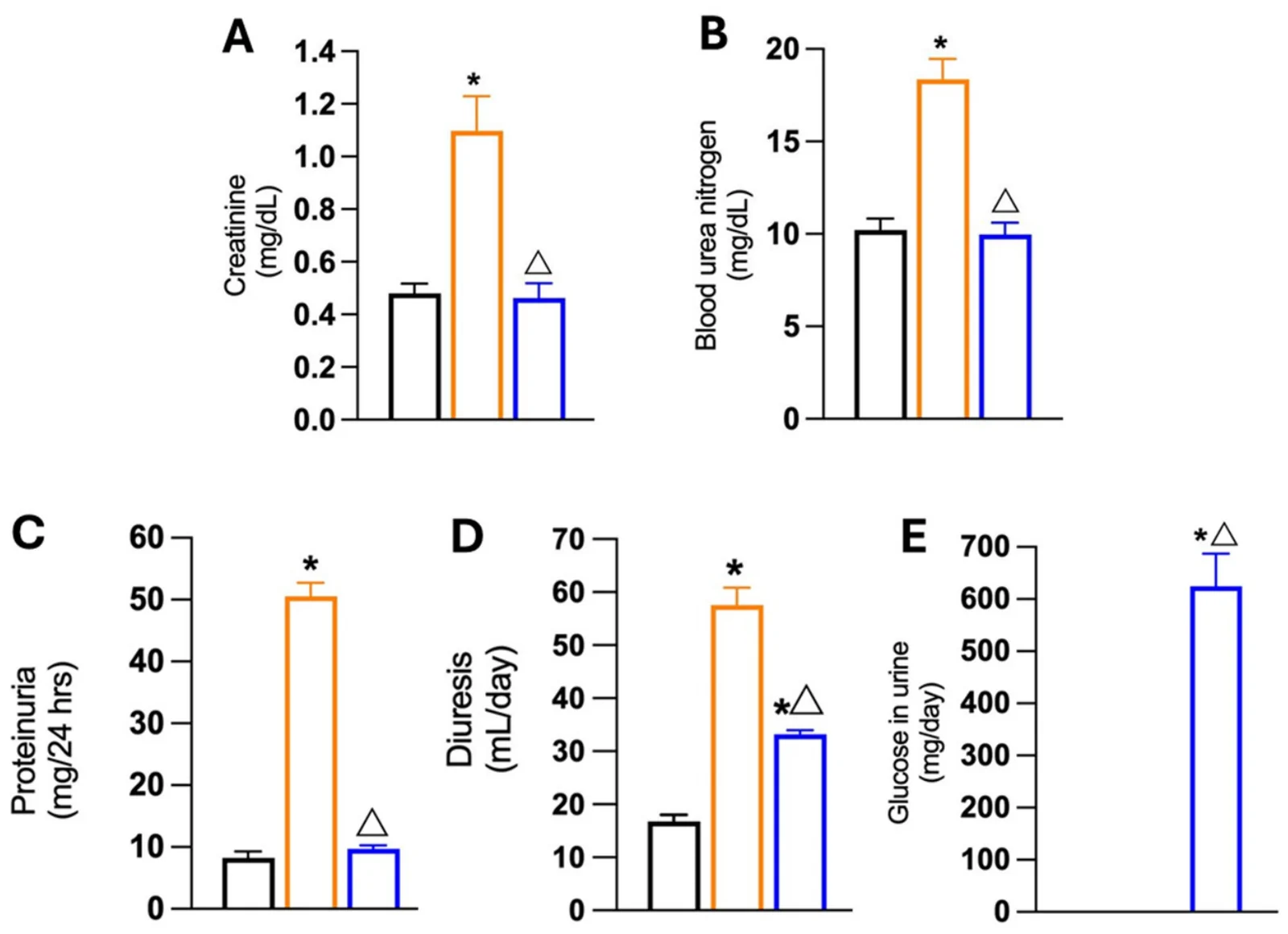
Effects of the empagliflozin+metformin combination on renal function in individuals with cardio–kidney–metabolic syndrome (CKMS). (**A**) Creatinine, (**B**) blood urea nitrogen, (**C**) proteinuria, (**D**) diuresis, and (**E**) glucosuria. Open bars in black lines (□) represent the control group (Ctrl), open bars in orange lines (□) correspond to the cardio–kidney–metabolic syndrome group (CKMS), and the bar in blue lines (□) represents the empagliflozin+metformin combination group (E+M). Data are expressed as mean ± SEM (n = 6) and analyzed by one-way ANOVA followed by Tukey’s multiple comparison test. * *p* < 0.05 vs. control; ^△^ *p* < 0.05 versus CKMS.

**Figure 6:**
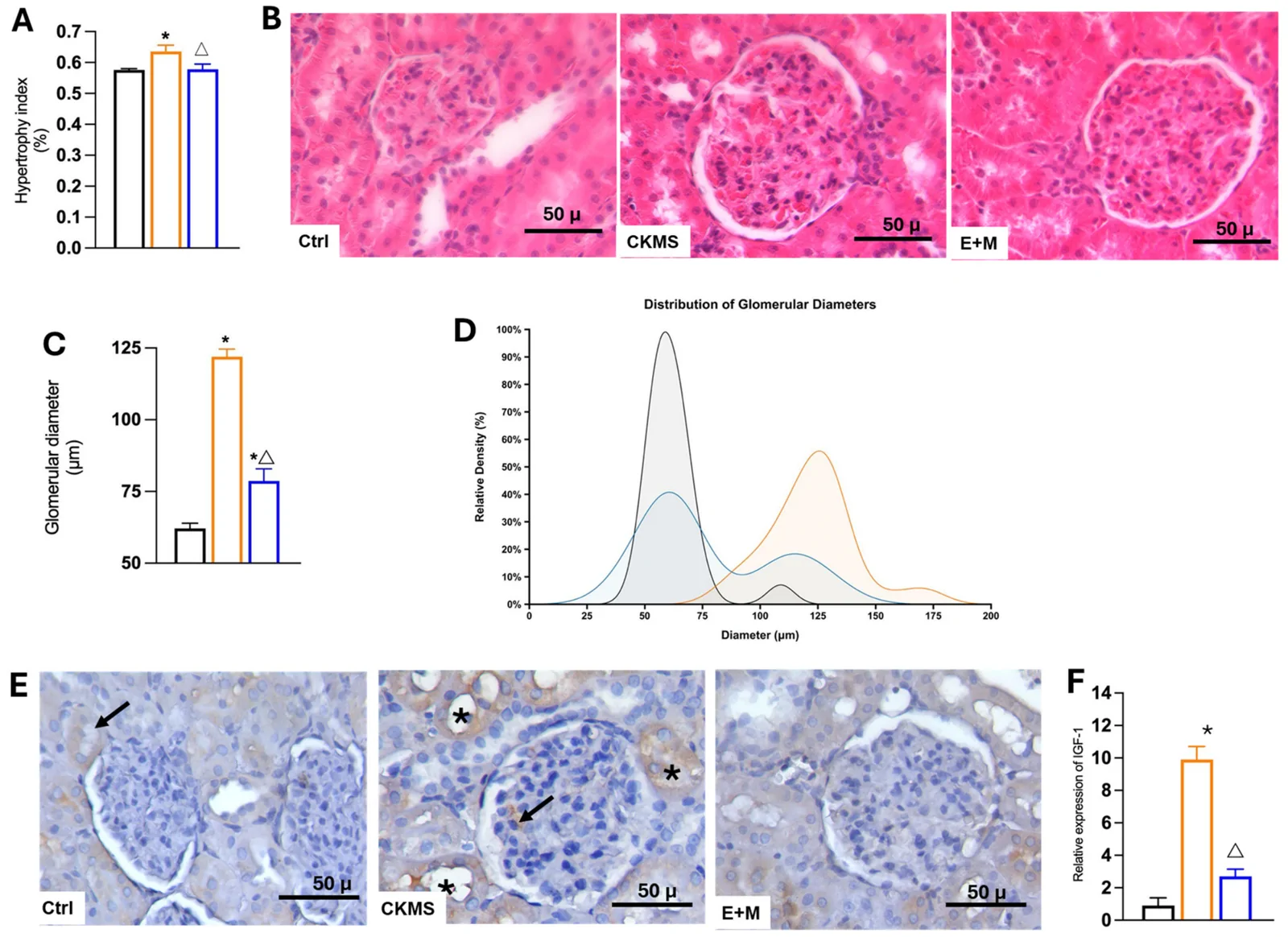
Histopathological analysis of the kidney. (**A**) Hypertrophy index and (**B**) representative micrographs of renal tissue stained with H&E from each experimental group, illustrating changes in glomerular morphology and diameter. Normal glomerular histology and size of control animal; in contrast, the CKMS group show larger hypercellular glomeruli. (**C**) Quantitative analysis of glomerular diameter and (**D**) kernel density estimation (KDE) plots illustrating the distribution of glomerular diameters across the control (black line) (□), CKMS (orange line) (□), and combined empagliflozin+metformin (blue line) (□) groups. Each curve represents the distribution of glomerular diameters, illustrating shifts in mean values, changes in dispersion, and multimodal distributions. (**E**) Representative micrographs of immunohistochemistry of insulin-like growth factor-1 (IGF-1) expression, control animals (Ctrl) that show weak IGF-1 immunostaining in some proximal cortical tubules (arrow), and CKMS cases that show stronger IGF-1 immunostaining in numerous proximal convoluted tubules (asterisk) and mesangial cells (arrow). Kidney sections from animals treated with empagliflozin+metformin (E+M) show normal glomerular cellularity and less proximal cortical tubules with reduced IGF-1 immunostaining. (**F**) Quantitative analysis of IGF-1 expression. For histopathological, immunohistochemical, and quantitative analyses, three animals per experimental group were randomly selected, and 20 glomeruli per sample were evaluated. Data are expressed as mean ± SEM and analyzed by one-way ANOVA followed by Tukey’s multiple comparison test. * *p* < 0.05 vs. control group; ^△^ *p* < 0.05 vs. CKMS group.

**Figure 7:**
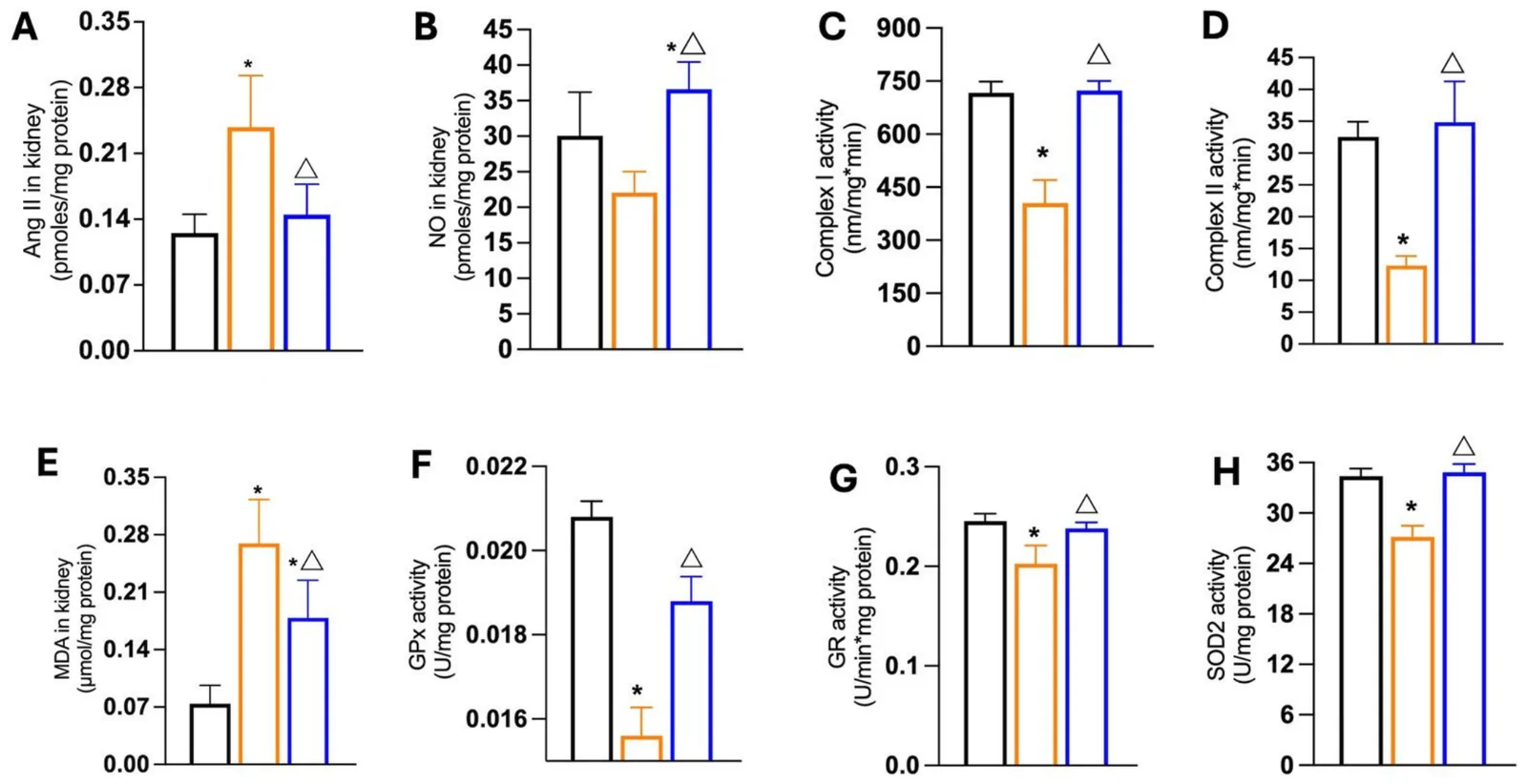
Effects of pharmacological treatment on vascular tone and oxidative stress in the renal cortex. (**A**) Angiotensin II and (**B**) nitric oxide (NO) concentrations, and (**C**) mitochondrial activity of complex I and (**D**) complex II, (**E**) lipid oxidation, (**F**) glutathione peroxidase activity, (**G**) glutathione reductase activity, and (**H**) superoxide dismutase 2 activity (SOD2). Open bars in black lines (□) represent the control group (Ctrl), open bars in orange lines (□) correspond to the cardio–kidney–metabolic syndrome group (CKMS), and the bar in blue lines (□) represents the empagliflozin+metformin combination group (E+M). Data are expressed as mean ± SEM (n = 6) and analyzed by one-way ANOVA followed by Tukey’s multiple comparison test. * *p* < 0.05 vs. control; ^Δ^ *p* < 0.05 vs. CKMS.

**Figure 8:**
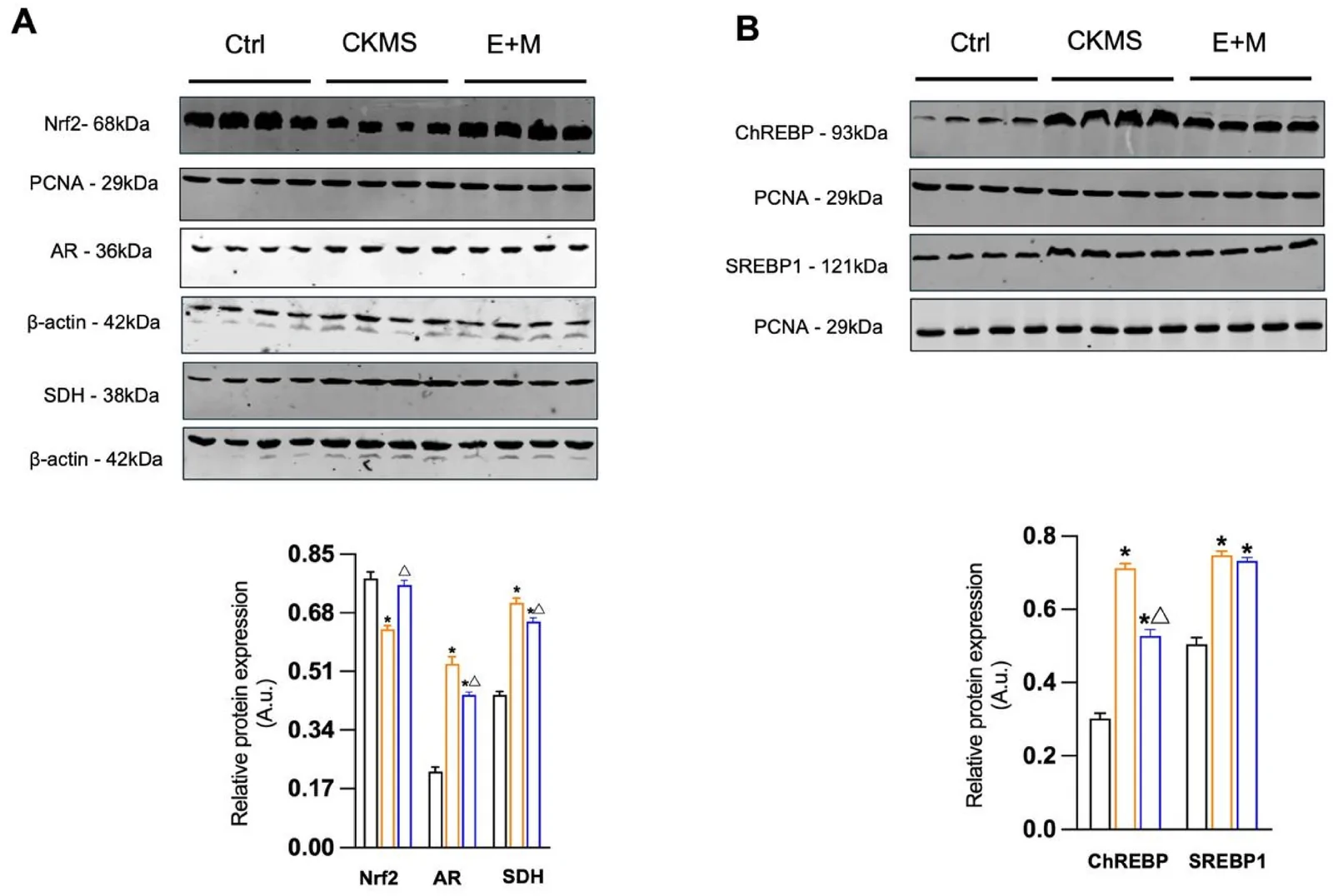
Effect of empagliflozin+metformin combination on the protein expression of (**A**) oxidative-stress related proteins and (**B**) transcription factors involved in the modulation of carbohydrate and lipid metabolism in the kidney cortex. Abbreviations: nuclear factor erythroid 2-related factor 2 (Nrf2), aldose reductase (AR), sorbitol dehydrogenase (SDH), carbohydrate-response element-binding protein (ChREBP), sterol regulatory element-binding protein (SREBP1), and proliferating cell nuclear antigen (PCNA). Open bars in black lines (□) represent the control group (Ctrl), open bars in orange lines (□) correspond to the cardio–kidney–metabolic syndrome group (CKMS), and the bar in blue lines (□) represents the empagliflozin+metformin combination group (E+M). For representative Western blotting, four randomly selected samples per group were analyzed. Data are expressed as mean ± SEM and analyzed by one-way ANOVA with Tukey’s multiple comparison tests. * *p* < 0.05 vs. control group; ^△^ *p* < 0.05 vs. CKMS group.

**Figure 9:**
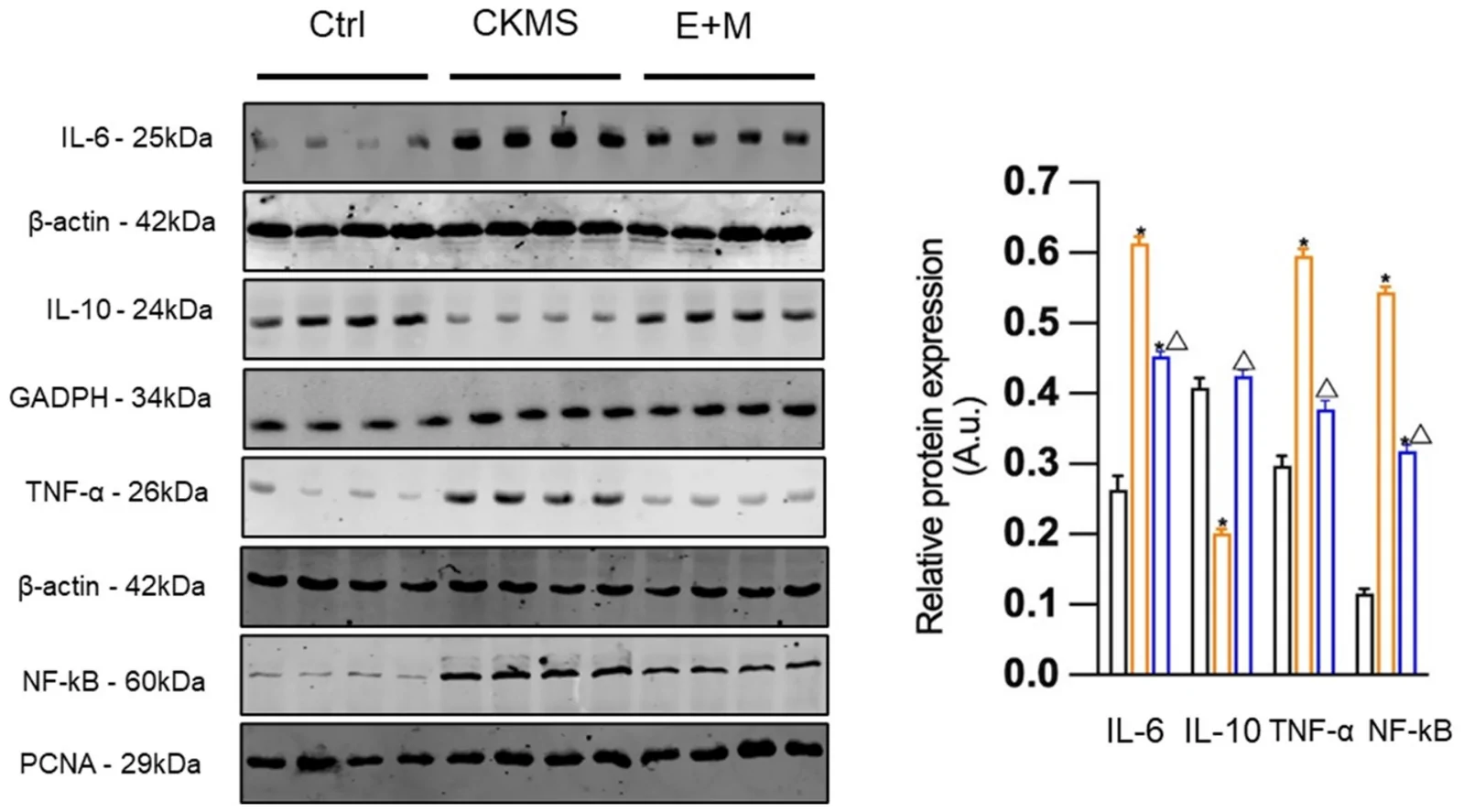
Effects of empagliflozin+metformin combination on the protein expression of inflammatory markers. Interleukin 6 (IL-6), interleukin 10 (IL-10), tumor necrosis factor-α (TNF-α), and nuclear factor kappa B (NF-κB) in the kidney cortex. Open bars in black lines (□) represent the control group (Ctrl), open bars in orange lines (**□**) correspond to the cardio–kidney–metabolic syndrome group (CKMS), and the bar in blue lines (**□**) represents the empagliflozin+metformin combination group (E+M). For representative Western blotting, four randomly selected samples per group were analyzed. Data are expressed as mean ± SEM and analyzed by one-way ANOVA with Tukey’s multiple comparison tests. * *p* < 0.05 vs. control group; ^△^ *p* < 0.05 vs. CKMS group.

**Table 1:** Metabolic profile and serum and urinary markers in the experimental groups, before pharmacological treatment.

	Parameter	Control	CKMS
	Body weight	266.9 ± 9.51	349.1 ± 3.46 *
Glycemic control	Blood glucose (mg/dL)	87.80 ± 2.24	106.4 ± 1.96 *
	OGTT [AUC (mg/dL/min)]	17,553 ± 560.68	21,204 ± 341.71 *
Lipid profile	Triglycerides (mg/dL)	51.12 ± 3.46	108.7 ± 5.72 *
	Total cholesterol (mg/dL)	52.74 ± 2.24	161.1 ± 8.27 *
	Plasma HDL-c (mg/dL)	40.42 ± 3.45	20.99 ± 1.70 *
	FFA (μM/L)	420.8 ± 39.98	1319 ± 62.02 *
Markers of cardiovascular risk and renal function	SBP (mmHg)	118.7 ± 1.97	146.8 ± 2.22 *
	Plasma UA (mg/dL)	1.41 ± 0.40	4.24 ±0.36 *
	Plasma creatinine (mg/dL)	0.44 ± 0.03	1 ± 0.11 *
	Urine UA (mg/dL)	1.58 ± 0.11	2.41 ± 0.10 *
	BUN (mg/dL)	13.61 ± 0.49	22.23 ± 0.94 *
	Proteinuria (mg/24 h)	11.09 ± 1.26	19.1 ± 1.17 *
	Diuresis (mL/24 h)	14 ± 1.48	38.8 ± 1.76 *

Abbreviations: area under the curve (AUC), blood urea nitrogen (BUN), control group (Ctrl), cardio–kidney–metabolic syndrome group (CKMS), free fatty acids (FFAs), high-density lipoprotein cholesterol (HDL-c), oral glucose tolerance test (OGTT), systolic blood pressure (SBP), and uric acid (UA). Data are presented as mean ± SEM; *t*-test of n = 6/control group and n = 12/CKMS group; statistical significance was established as * *p* < 0.05 vs. control group.

## Data Availability

The original contributions presented in this study are included in the article. Further inquiries can be directed to the corresponding authors.

## Supplementary Materials

The following supporting information can be downloaded at: https://www.mdpi.com/article/10.3390/ijms27208988/s1.

## Abbreviations

The following abbreviations are used in this manuscript:

ACC	Acetyl-CoA carboxylase
ALP	Alkaline phosphatase
ALT	Alanine transaminase
AMP	AMP-activated protein kinase
AMPK	(AMP)-activated protein kinase alpha
ANG II	Angiotensin II
AOPPs	Advanced oxidation protein products
AR	Aldose reductase
AST	Aspartate aminotransferase
ATP	Adenosine triphosphate
BT	Bilirubin total
BUN	Blood urea nitrogen
CKMS	Cardio–kidney–metabolic syndrome
ChREBP	Carbohydrate-response element-binding protein
CKD	Chronic kidney disease
Ctrl	Control group
CV	Cardiovascular
DB	Direct bilirubin
DNL	De novo lipogenesis
DNPH	2,4-dinitrophenylhydrazine
FASN	Fatty acid synthase
FFA	Free fatty acid
FGB	Fasting blood glucose
G6P	Glucose-6-phosphate
GGT	γ-Glutamyl transferase
GLP-1	Glucagon-like peptide 1 agonist
GPx	Glutathione peroxidase
GR	Glutathione reductase
H&E	Hematoxylin and eosin stain
HDL-c	High-density lipoprotein cholesterol
HEC	Hydroxyethyl cellulose
HFD	High-fat diet
IGF-1	Insulin-like growth factor 1
IL	Interleukin
IL-6	Interleukin-6
IR	Insulin resistance
KDE	Kernel density estimation
LVDD	Left ventricular diastolic dysfunction
LXR	Liver X receptor alpha
MASLD	Metabolic dysfunction-associated steatotic liver disease
MDA	Malondialdehyde
MetS	Metabolic syndrome
NaOH	Sodium hydroxide
NASH	Non-alcoholic steatohepatitis
NAFLD	Non-alcoholic fatty liver disease
NF-κB	Nuclear factor kappa B
NOX	NADPH oxidase
NLRP3	Nucleotide-binding domain, leucine-rich repeat-containing family, pyrin domain containing 3
Nrf2	Nuclear factor erythroid 2-related factor 2
OGTT	Oral Glucose Tolerance Test
ON	Nitric oxide
PBS	Phosphate-buffered saline
PPAR	Peroxisome proliferator-activated receptor
PTW	Thickness the posterior wall
PUFAs	Polyunsaturated fatty acid
RAAS	Renin angiotensin aldosterone system
ROS	Reactive oxygen species
SBP	Systolic blood pressure
SDH	Sorbitol dehydrogenase
SEM	Standard error of the mean
SGLT2	Sodium–glucose co-transporter 2
SGLT2i	Sodium–Glucose co-transporter type 2 inhibitor
SOD	Superoxide dismutase
SREBP-1c	Sterol regulatory element-binding protein 1c
T2DM	Type 2 diabetes mellitus
TAS	Total antioxidant status
TC	Total cholesterol
TGs	Triglycerides
TNF-alpha	Tumor necrosis factor-alpha
UA	Uric acid
VLDL	Very low-density lipoprotein
VSMC	Vascular smooth muscle cell
ACC	Acetyl-CoA carboxylase
ALP	Alkaline phosphatase
ALT	Alanine transaminase
AMP	AMP-activated protein kinase

## References

[1] Ferdinand K.C. (2024). An overview of cardiovascular-kidney-metabolic syndrome. Am. J. Manag. Care.

[2] Tsai M.K., Kao J.T., Wong C.S., Liao C.T., Lo W.C., Chien K.L., Wen C.P., Wu M.S., Wu M.Y. (2025). Cardiovascular-kidney-metabolic syndrome and all-cause and cardiovascular mortality: A retrospective cohort study. PLoS Med..

[3] Wang H.H., Lee D.K., Liu M., Portincasa P., Wang D.Q. (2020). Novel Insights into the Pathogenesis and Management of the Metabolic Syndrome. Pediatr. Gastroenterol. Hepatol. Nutr..

[4] Bovolini A., Garcia J., Andrade M.A., Duarte J.A. (2021). Metabolic Syndrome Pathophysiology and Predisposing Factors. Int. J. Sports Med..

[5] Arellano Buendia A.S., Juárez Rojas J.G., García-Arroyo F., Aparicio Trejo O.E., Sánchez-Muñoz F., Argüello-García R., Sánchez-Lozada L.G., Bojalil R., Osorio-Alonso H. (2023). Antioxidant and anti-inflammatory effects of allicin in the kidney of an experimental model of metabolic syndrome. PeerJ.

[6] Singh A., Kesani H., Verma S., Saleh T.M., Rai M. (2025). Cardio-Renal Metabolic Syndrome: An Integrated Approach to Prevention and Management. Cureus.

[7] Han Y., Yao M., Zhao H., Han X., Di H., Xie T., Wu J., Wang Y., Zhang Y., Zeng X. (2025). Exploration of the Interrelationship Between Serum Uric Acid, Gout, and Cardiac, Renal, and Metabolic Conditions in Middle Aged and Older People. J. Am. Heart Assoc..

[8] Bowden R.G., Richardson K.A., Richardson L.T. (2022). Uric acid and metabolic syndrome: Findings from national health and nutrition examination survey. Front. Med..

[9] Du L., Zong Y., Li H., Wang Q., Xie L., Yang B., Pang Y., Zhang C., Zhong Z., Gao J. (2024). Hyperuricemia and its related diseases: Mechanisms and advances in therapy. Signal Transduct. Target. Ther..

[10] Tanaka Y., Nagoshi T., Kawai M., Uno G., Ito S., Yoshii A., Kimura H., Inoue Y., Ogawa K., Tanaka T.D. (2017). Close linkage between serum uric acid and cardiac dysfunction in patients with ischemic heart disease according to covariance structure analysis. Sci. Rep..

[11] Petreski T., Ekart R., Hojs R., Bevc S. (2020). Hyperuricemia, the heart, and the kidneys-to treat or not to treat?. Ren. Fail..

[12] Abdul-Ghani M.A., Matsuda M., Jani R., Jenkinson C.P., Coletta D.K., Kaku K., DeFronzo R.A. (2008). The relationship between fasting hyperglycemia and insulin secretion in subjects with normal or impaired glucose tolerance. Am. J. Physiol. Endocrinol. Metab..

[13] Yan L.J. (2018). Redox imbalance stress in diabetes mellitus: Role of the polyol pathway. Anim. Model. Exp. Med..

[14] Copur S., Demiray A., Kanbay M. (2022). Uric acid in metabolic syndrome: Does uric acid have a definitive role?. Eur. J. Intern. Med..

[15] Tu Y.C., Liu Y.H., Chen S.C., Su H.M. (2023). Metabolic Syndrome and Its Components Are Associated with New-Onset Hyperuricemia in a Large Taiwanese Population Follow-Up Study. Nutrients.

[16] Wei X., Zhang M., Huang S., Lan X., Zheng J., Luo H., He Y., Lei W. (2023). Hyperuricemia: A key contributor to endothelial dysfunction in cardiovascular diseases. FASEB J..

[17] Bhatti J.S., Bhatti G.K., Reddy P.H. (2017). Mitochondrial dysfunction and oxidative stress in metabolic disorders—A step towards mitochondria based therapeutic strategies. Biochim. Biophys. Acta Mol. Basis Dis..

[18] Kleibert M., Zygmunciak P., Łakomska K., Mila K., Zgliczyński W., Mrozikiewicz-Rakowska B. (2023). Insight into the Molecular Mechanism of Diabetic Kidney Disease and the Role of Metformin in Its Pathogenesis. Int. J. Mol. Sci..

[19] Lin L., Tan W., Pan X., Tian E., Wu Z., Yang J. (2022). Metabolic Syndrome-Related Kidney Injury: A Review and Update. Front. Endocrinol..

[20] Xu Z., Yang S., Tan Y., Zhang Q., Wang H., Tao J., Liu Q., Wang Q., Feng W., Li Z. (2025). Inflammation in cardiovascular-kidney-metabolic syndrome: Key roles and underlying mechanisms-a comprehensive review. Mol. Cell Biochem..

[21] Gao C., Gao S., Zhao R., Shen P., Zhu X., Yang Y., Duan C., Wang Y., Ni H., Zhou L. (2024). Association between systemic immune-inflammation index and cardiovascular-kidney-metabolic syndrome. Sci. Rep..

[22] Yao Z., Dardari Z.A., LaMonte M.J., Matsushita K., Ballantyne C.M., Lima J.A., Vasan R.S., Simonsick E.M., Appel L.J., Cohen D.L. (2026). The Prognostic Value of Serum Interleukin-6 Concentrations for 9 Cardiovascular and Mortality Outcomes. JACC.

[23] Ridker P.M., MacFadyen J.G., Glynn R.J., Koenig W., Libby P., Everett B.M., Lefkowitz M., Thuren T., Cornel J.H. (2018). Inhibition of Interleukin-1β by Canakinumab and Cardiovascular Outcomes in Patients With Chronic Kidney Disease. J. Am. Coll. Cardiol..

[24] Cortés-Camacho F., Zambrano-Vásquez O.R., Aréchaga-Ocampo E., Castañeda-Sánchez J.I., Gonzaga-Sánchez J.G., Sánchez-Gloria J.L., Sánchez-Lozada L.G., Osorio-Alonso H. (2024). Sodium-Glucose Cotransporter Inhibitors: Cellular Mechanisms Involved in the Lipid Metabolism and the Treatment of Chronic Kidney Disease Associated with Metabolic Syndrome. Antioxid..

[25] Piwkowska A., Rogacka D., Jankowski M., Dominiczak M.H., Stepiński J.K., Angielski S. (2010). Metformin induces suppression of NAD(P)H oxidase activity in podocytes. Biochem. Biophys. Res. Commun..

[26] Zhang S., Xu H., Yu X., Wu Y., Sui D. (2017). Metformin ameliorates diabetic nephropathy in a rat model of low-dose streptozotocin-induced diabetes. Exp. Ther. Med..

[27] Laiteerapong N., Ham S.A., Gao Y., Moffet H.H., Liu J.Y., Huang E.S., Karter A.J. (2019). The Legacy Effect in Type 2 Diabetes: Impact of Early Glycemic Control on Future Complications (The Diabetes & Aging Study). Diabetes Care.

[28] Caturano A., Galiero R., Loffredo G., Vetrano E., Medicamento G., Acierno C., Rinaldi L., Marrone A., Salvatore T., Monda M. (2023). Effects of a Combination of Empagliflozin Plus Metformin vs. Metformin Monotherapy on NAFLD Progression in Type 2 Diabetes: The IMAGIN Pilot Study. Biomedicines.

[29] Kambham N., Markowitz G.S., Valeri A.M., Lin J., D’Agati V.D. (2001). Obesity-related glomerulopathy: An emerging epidemic. Kidney Int..

[30] Frystyk J., Skjaerbaek C., Vestbo E., Fisker S., Orskov H. (1999). Circulating levels of free insulin-like growth factors in obese subjects: The impact of type 2 diabetes. Diabetes Metab. Res. Rev..

[31] Janić M., Cankar M., Šmid J., France Štiglic A., Jerin A., Šabovič M., Janež A., Lunder M. (2022). Empagliflozin-Metformin Combination Has Antioxidative and Anti-Inflammatory Properties that Correlate with Vascular Protection in Adults with Type 1 Diabetes. J. Diabetes Res..

[32] Caturano A., Galiero R., Simeon V., Acierno C., Nilo D., Marrone A., Trotta M.C., Vetrano E., Sardu C., Russo V. (2025). Impact of empagliflozin as add-on to metformin vs metformin continuation on MASLD progression in type 2 diabetes: The IMAGIN Study. Nutr. Metab. Cardiovasc. Dis..

[33] Madhag Z., Al-Isawi Z. (2024). Empagliflozin alone and in combination with metformin mitigates diabetes-associated renal complications. J. Med. Life.

[34] Häring H.U., Merker L., Seewaldt-Becker E., Weimer M., Meinicke T., Broedl U.C., Woerle H.J. (2014). Empagliflozin as add-on to metformin in patients with type 2 diabetes: A 24-week, randomized, double-blind, placebo-controlled trial. Diabetes Care.

[35] Russo E., Bertolotto M., Zanetti V., Picciotto D., Esposito P., Carbone F., Montecucco F., Pontremoli R., Garibotto G., Viazzi F. (2023). Role of Uric Acid in Vascular Remodeling: Cytoskeleton Changes and Migration in VSMCs. Int. J. Mol. Sci..

[36] Sanchez-Lozada L.G., Soto V., Tapia E., Avila-Casado C., Sautin Y.Y., Nakagawa T., Franco M., Rodriguez-Iturbe B., Johnson R.J. (2008). Role of oxidative stress in the renal abnormalities induced by experimental hyperuricemia. Am. J. Physiol. Ren. Physiol..

[37] Bogoviku J., Nguyen T.D., Westphal J.G., Haertel F., Aftanski P., Möbius-Winkler S., Busch M., Schulze P.C. (2025). Effects of empagliflozin on uric acid levels during acute heart failure recompensation: A sub-analysis of the EMPAG-HF trial (Effects of Empagliflozin on Diuresis and Renal Function in Patients with Acute Decompensated Heart Failure). Eur. J. Heart Fail..

[38] Vuraloğlu E., Kut A., Turhan İyidir Ö. (2025). Effect of Glomerular Filtration Rate on Uric Acid Metabolism in a Retrospective Cohort of Type-2 Diabetes Mellitus Patients on SGLT-2 Inhibitor Therapy. J. Clin. Pract. Res..

[39] Zhang L., Zhang F., Bai Y., Huang L., Zhong Y., Zhang X. (2024). Effects of sodium-glucose cotransporter-2 (SGLT-2) inhibitors on serum uric acid levels in patients with chronic kidney disease: A systematic review and network meta-analysis. BMJ Open Diabetes Res. Care.

[40] Zhang G., Ma Y., Xi D., Rao Z., Sun X., Wu X. (2019). Effect of high uric acid on the disposition of metformin: In vivo and in vitro studies. Biopharm. Drug Dispos..

[41] Ahwin P., Martinez D. (2024). The relationship between SGLT2 and systemic blood pressure regulation. Hypertens. Res..

[42] Griffin M., Rao V.S., Ivey-Miranda J., Fleming J., Mahoney D., Maulion C., Suda N., Siwakoti K., Ahmad T., Jacoby D. (2020). Empagliflozin in Heart Failure: Diuretic and Cardiorenal Effects. Circulation.

[43] Díaz-Cruz C., González-Ortiz M., Rosales-Rivera L.Y., Patiño-Laguna A.J., Ramírez-Rodríguez Z.G., Díaz-Cruz K., Martínez-Abundis E. (2020). Effects of dapagliflozin on blood pressure variability in patients with prediabetes and prehypertension without pharmacological treatment: A randomized trial. Blood Press. Monit..

[44] Hamidi Shishavan M., Henning R.H., van Buiten A., Goris M., Deelman L.E., Buikema H. (2017). Metformin Improves Endothelial Function and Reduces Blood Pressure in Diabetic Spontaneously Hypertensive Rats Independent from Glycemia Control: Comparison to Vildagliptin. Sci. Rep..

[45] Drzewoski J., Hanefeld M. (2021). The Current and Potential Therapeutic Use of Metformin—The Good Old Drug. Pharmaceuticals.

[46] Sabbar R., Kadhim S.A.A., Fawzi H.A., Flayih A., Mohammad B., Swadi A. (2024). The impact of empagliflozin and metformin on cardiac parameters in patients with mid-range ejection fraction heart failure without diabetes. J. Med. Life.

[47] Prochaska J.H., Jünger C., Schulz A., Arnold N., Müller F., Heidorn M.W., Baumkötter R., Zahn D., Koeck T., Tröbs S.O. (2023). Effects of empagliflozin on left ventricular diastolic function in addition to usual care in individuals with type 2 diabetes mellitus-results from the randomized, double-blind, placebo-controlled EmDia trial. Clin. Res. Cardiol..

[48] Doehner W., Anker S.D., Butler J., Zannad F., Filippatos G., Ferreira J.P., Salsali A., Kaempfer C., Brueckmann M., Pocock S.J. (2022). Uric acid and sodium-glucose cotransporter-2 inhibition with empagliflozin in heart failure with reduced ejection fraction: The EMPEROR-reduced trial. Eur. Heart J..

[49] Bazoukis G., Papadatos S.S., Thomopoulos C., Tse G., Cheilidis S., Tsioufis K., Farmakis D. (2021). Impact of SGLT2 inhibitors on major clinical events and safety outcomes in heart failure patients: A meta-analysis of randomized clinical trials. J. Geriatr. Cardiol..

[50] Jiang K., Su F., Deng R., Xu Y., Qin A., Yuan X., Xing D., Chen Y., Wang D., Shen L. (2025). Cardiomyocyte-specific NHE1 overexpression confers protection against myocardial infarction during hyperglycemia. Cardiovasc. Diabetol..

[51] Endo S., Kanamori H., Yoshida A., Naruse G., Komura S., Minatoguchi S., Watanabe T., Kawaguchi T., Yamada Y., Mikami A. (2025). Sodium-glucose cotransporter 2 inhibitor empagliflozin enhances autophagy and reverses remodeling in hearts with large, old myocardial infarctions. Eur. J. Pharmacol..

[52] Chen S., Wang Q., Bakker D., Hu X., Zhang L., van der Made I., Tebbens A.M., Kovácsházi C., Giricz Z., Brenner G.B. (2024). Empagliflozin prevents heart failure through inhibition of the NHE1-NO pathway, independent of SGLT2. Basic. Res. Cardiol..

[53] Rutledge C.A., Kaufman B.A., Dezfulian C., Elmer J. (2022). Metformin protects against cardiac and renal damage in diabetic cardiac arrest patients. Resuscitation.

[54] Zamami R., Kohagura K., Kinjyo K., Nakamura T., Kinjo T., Yamazato M., Ishida A., Ohya Y. (2021). The Association between Glomerular Diameter and Secondary Focal Segmental Glomerulosclerosis in Chronic Kidney Disease. Kidney Blood Press. Res..

[55] Contreras C., Muñoz M., Freire-Agulleiro Ó., Estévez Á., Martínez M.P., Olmos L., Gómez del Val A., Rodríguez C., Silvestre R.A., Sánchez A. (2025). Obesity-induced arterial redox imbalance involving mitochondrial NOX4, endothelial dysfunction, and ER stress underlie kidney injury compensated by enhanced mitochondrial bioenergetics. Redox Biol..

[56] Gurevich E., Segev Y., Landau D. (2021). Growth Hormone and IGF1 Actions in Kidney Development and Function. Cells.

[57] Flyvbjerg A., Bornfeldt K.E., Marshall S.M., Arnqvist H.J., Orskov H. (1990). Kidney IGF-I mRNA in initial renal hypertrophy in experimental diabetes in rats. Diabetologia.

[58] Greka A., Mundel P. (2012). Cell biology and pathology of podocytes. Annu. Rev. Physiol..

[59] Du S.J., Huang W., Hao Y., Zhang C., He F.F. (2025). Mechanical Stress and Protective Mechanisms in Podocytes: Insights into Hypertensive Nephropathy. Int. J. Mol. Sci..

[60] Sen T., Heerspink H.J.L. (2021). A kidney perspective on the mechanism of action of sodium glucose co-transporter 2 inhibitors. Cell Metab..

[61] Rogacka D., Piwkowska A. (2021). Beneficial effects of metformin on glomerular podocytes in diabetes. Biochem. Pharmacol..

[62] Liu B., Fan Z., Edgerton S.M., Yang X., Lind S.E., Thor A.D. (2011). Potent anti-proliferative effects of metformin on trastuzumab-resistant breast cancer cells via inhibition of erbB2/IGF-1 receptor interactions. Cell Cycle.

[63] Yang C.C., Yeh J.N., Hsu T.W., Chiang J.Y., Cheng B.C., Sung P.H., Wu S.C., Huang C.R., Shao P.L., Yip H.K. (2025). Empagliflozin protected kidney function in CKD rat through suppressing hypoxic and fibrotic signalings mediated inflammation and EMT. Histol. Histopathol..

[64] Mohebi R., Liu Y., Hansen M.K., Yavin Y., Sattar N., Pollock C.A., Butler J., Jardine M., Masson S., Heerspink H.J.L. (2023). Insulin growth factor axis and cardio-renal risk in diabetic kidney disease: An analysis from the CREDENCE trial. Cardiovasc. Diabetol..

[65] Vasilkova V.N., Mokhort T.V., Pchelin I.Y., Bayrasheva V.K., Naumenko E.P., Korotaeva L.E., Filiptsova N.A. (2021). Association between serum insulin like growth factor-1 (IGF-1) and insulin-like growth factor-binding protein-3 levels and chronic kidney disease in diabetic patients. J. Ren. Inj. Prev..

[66] Lunder M., Janić M., Japelj M., Juretič A., Janež A., Šabovič M. (2018). Empagliflozin on top of metformin treatment improves arterial function in patients with type 1 diabetes mellitus. Cardiovasc. Diabetol..

[67] Afzal S., Abdul Manap A.S., Attiq A., Albokhadaim I., Kandeel M., Alhojaily S.M. (2023). From imbalance to impairment: The central role of reactive oxygen species in oxidative stress-induced disorders and therapeutic exploration. Front. Pharmacol..

[68] Balaban R.S., Mandel L.J. (1988). Metabolic substrate utilization by rabbit proximal tubule. An NADH fluorescence study. Am. J. Physiol..

[69] Aparicio-Trejo O.E., Avila-Rojas S.H., Tapia E., Rojas-Morales P., León-Contreras J.C., Martínez-Klimova E., Hernández-Pando R., Sánchez-Lozada L.G., Pedraza-Chaverri J. (2020). Chronic impairment of mitochondrial bioenergetics and β-oxidation promotes experimental AKI-to-CKD transition induced by folic acid. Free Radic. Biol. Med..

[70] Wang Y., Zhang X., Yao H., Chen X., Shang L., Li P., Cui X., Zeng J. (2022). Peroxisome-generated succinate induces lipid accumulation and oxidative stress in the kidneys of diabetic mice. J. Biol. Chem..

[71] Lubawy M., Formanowicz D. (2023). High-Fructose Diet–Induced Hyperuricemia Accompanying Metabolic Syndrome–Mechanisms and Dietary Therapy Proposals. Int. J. Environ. Res. Public Health.

[72] Hotta N., Kawamura T., Umemura T. (2020). Are the polyol pathway and hyperuricemia partners in the development of non-alcoholic fatty liver disease in diabetes?. J. Diabetes Investig..

[73] Wakil S.J., Abu-Elheiga L.A. (2009). Fatty acid metabolism: Target for metabolic syndrome. J. Lipid Res..

[74] Esler W.P., Bence K.K. (2019). Metabolic Targets in Nonalcoholic Fatty Liver Disease. Cell Mol. Gastroenterol. Hepatol..

[75] Yaribeygi H., Maleki M., Reiner Ž., Jamialahmadi T., Sahebkar A. (2022). Mechanistic View on the Effects of SGLT2 Inhibitors on Lipid Metabolism in Diabetic Milieu. J. Clin. Med..

[76] Zhou G., Myers R., Li Y., Chen Y., Shen X., Fenyk-Melody J., Wu M., Ventre J., Doebber T., Fujii N. (2001). Role of AMP-activated protein kinase in mechanism of metformin action. J. Clin. Investig..

[77] Uyeda K., Repa J.J. (2006). Carbohydrate response element binding protein, ChREBP, a transcription factor coupling hepatic glucose utilization and lipid synthesis. Cell Metab..

[78] Kawaguchi T., Takenoshita M., Kabashima T., Uyeda K. (2001). Glucose and cAMP regulate the L-type pyruvate kinase gene by phosphorylation/dephosphorylation of the carbohydrate response element binding protein. Proc. Natl. Acad. Sci. USA.

[79] Shi F., Collins S. (2023). Regulation of mTOR Signaling: Emerging Role of Cyclic Nucleotide-Dependent Protein Kinases and Implications for Cardiometabolic Disease. Int. J. Mol. Sci..

[80] Bravo-Ruiz I., Medina M., Martínez-Poveda B. (2021). From Food to Genes: Transcriptional Regulation of Metabolism by Lipids and Carbohydrates. Nutrients.

[81] Dentin R., Tomas-Cobos L., Foufelle F., Leopold J., Girard J., Postic C., Ferré P. (2012). Glucose 6-phosphate, rather than xylulose 5-phosphate, is required for the activation of ChREBP in response to glucose in the liver. J. Hepatol..

[82] Rispoli R.M., Popolo A., De Fabrizio V., d’Emmanuele di Villa Bianca R., Autore G., Dalli J., Marzocco S. (2025). Targeting Inflammatory Imbalance in Chronic Kidney Disease: Focus on Anti-Inflammatory and Resolution Mediators. Int. J. Mol. Sci..

[83] Tang S.C.W., Yiu W.H. (2020). Innate immunity in diabetic kidney disease. Nat. Rev. Nephrol..

[84] Amorim R.G., Guedes G.D.S., Vasconcelos S.M.L., Santos J.C.F. (2019). Kidney Disease in Diabetes Mellitus: Cross-Linking between Hyperglycemia, Redox Imbalance and Inflammation. Arq. Bras. Cardiol..

[85] Efiong E.E., Maedler K., Effa E., Osuagwu U.L., Peters E., Ikebiuro J.O., Soremekun C., Ihediwa U., Niu J., Fuchs M. (2025). Decoding diabetic kidney disease: A comprehensive review of interconnected pathways, molecular mediators, and therapeutic insights. Diabetol. Metab. Syndr..

[86] Wada J., Makino H. (2013). Inflammation and the pathogenesis of diabetic nephropathy. Clin. Sci..

[87] Navarro-González J.F., Mora-Fernández C. (2008). The role of inflammatory cytokines in diabetic nephropathy. J. Am. Soc. Nephrol..

[88] Thomas M.C., Brownlee M., Susztak K., Sharma K., Jandeleit-Dahm K.A., Zoungas S., Rossing P., Groop P.H., Cooper M.E. (2015). Diabetic kidney disease. Nat. Rev. Dis. Primers.

[89] Morgan M.J., Liu Z.G. (2011). Crosstalk of reactive oxygen species and NF-κB signaling. Cell Res..

[90] Reuter S., Gupta S.C., Chaturvedi M.M., Aggarwal B.B. (2010). Oxidative stress, inflammation, and cancer: How are they linked?. Free Radic. Biol. Med..

[91] Moore K.W., de Waal Malefyt R., Coffman R.L., O’Garra A. (2001). Interleukin-10 and the interleukin-10 receptor. Annu. Rev. Immunol..

[92] Zambrano-Vásquez O.R., Cortes-Camacho F., Cabrera-Angeles J.C., Hernández-Alba A.L., García-Arroyo F.E., Castañeda-Sánchez J.I., Aréchaga-Ocampo E., Aparicio-Trejo O.E., Valle-Mondragón L.D., Martínez-Olivares C.E. (2025). The Combination Empagliflozin/Metformin Attenuates the Progression of Metabolic Dysfunction-Associated Steatotic Liver Disease in a Diet-Induced Experimental Rat Model. Int. J. Mol. Sci..

[93] Jaikumkao K., Thongnak L., Htun K.T., Pengrattanachot N., Phengpol N., Sutthasupha P., Promsan S., Montha N., Sriburee S., Kothan S. (2024). Dapagliflozin and metformin in combination ameliorates diabetic nephropathy by suppressing oxidative stress, inflammation, and apoptosis and activating autophagy in diabetic rats. Biochim. Biophys. Acta Mol. Basis Dis..

[94] Hadjadj S., Rosenstock J., Meinicke T., Woerle H.J., Broedl U.C. (2016). Initial Combination of Empagliflozin and Metformin in Patients With Type 2 Diabetes. Diabetes Care.

[95] Sánchez-Gloria J.L., Martínez-Olivares C.E., Del Valle-Mondragón L., Cortés-Camacho F., Zambrano-Vásquez O.R., Hernández-Pando R., Sánchez-Muñoz F., Sánchez-Lozada L.G., Osorio-Alonso H. (2023). Allicin, an Emerging Treatment for Pulmonary Arterial Hypertension: An Experimental Study. Int. J. Mol. Sci..

[96] Varela-López E., Del Valle-Mondragón L., Castrejón-Téllez V., Pérez-Torres I., Arenas A.P., Rojas F.M., Guarner-Lans V., Vargas-González A., Pastelín-Hernández G., Torres-Narváez J.C. (2021). Role of the Transient Receptor Potential Vanilloid Type 1 (TRPV1) in the Regulation of Nitric Oxide Release in Wistar Rat Aorta. Oxid. Med. Cell Longev..

