# Supplementary material for: Empagliflozin and Metformin Combination Therapy Attenuates Oxidative Stress and Inflammation in Experimental Cardio–Kidney–Metabolic Syndrome
Source: Int J Mol Sci. 2026 Oct 10;27(20):8988. doi: 10.3390/ijms27208988 (PMC13704620; doi:10.3390/ijms27208988)
Supplement: Supplementary file 1 [file ijms-27-08988-s001.zip › ijms-4513554-supplementary.pdf]

Supplementary file

To evaluate glomerular and tubular kidney injury and its response to therapy, we analyzed the protein expression of synaptopodin, as a glomerular marker and kidney injury molecule-1 (KIM-1) and claudin 2 as biomarkers of tubular damage.

First, we analyzed the renal protein expression of synaptopodin, a critical protein essential for maintaining podocyte cytoskeletal architecture. The Control group exhibited basal levels of synaptopodin protein expression. In contrast, the CKMS group displayed a significant upregulation of synaptopodin (Supplementary figure 1, p<0.05 vs. Control group), reflecting alterations in podocyte cytoskeletal dynamics induced by the dysfunctional cardiometabolic environment. Notably, combination treatment with E+M significantly attenuated the upregulation of synaptopodin protein expression compared with the untreated CKMS group (Supplementary figure 1, p<0.05 vs. CKMS group). These results demonstrate that E+M combination therapy effectively prevents CKMS-induced podocyte alterations and restores renal synaptopodin expression toward basal conditions.

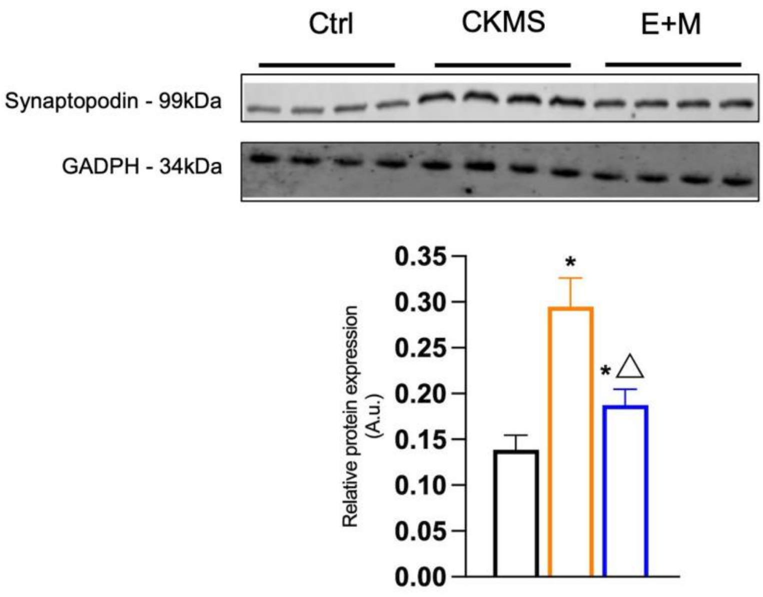

Figure S1. Effect of E+M combination treatment on glomerular injury marker synaptopodin. Representative immunoblot and densitometric analysis of synaptopodin protein relative expression normalized to GAPDH. Open bars in black lines (□) represent the control group (Ctrl), and open bars in orange lines (□) correspond to the cardio-kidney metabolic syndrome group (CKMS), and the bar in blue lines (□) represents the combination empagliflozin+metformin group (E+M). Data are expressed as mean ± SEM (n=4 biologically independent samples per group) and analyzed by one-way ANOVA with Tukey's multiple comparison tests. *p < 0.05 vs control group; △p < 0.05 vs CKMS group.

Second, the protein expression of KIM-1 was assessed. Control group presented basal and barely detectable levels of KIM-1 protein expression, reflecting normal tubular integrity. In contrast, the CKMS group exhibited a dramatic and significant increase (Supplementary figure 2, $p < 0.05$ vs Control group), indicating marked tubular injury induced by the

dysfunctional cardiometabolic environment. Notably, combination treatment with E+M significantly attenuated the upregulation of KIM-1 protein expression compared with the untreated CKMS group (Supplementary figure 2, $p < 0.05$ vs CKMS group). These results demonstrate that E+M combination therapy effectively reduces renal tubular damage under CKMS conditions.

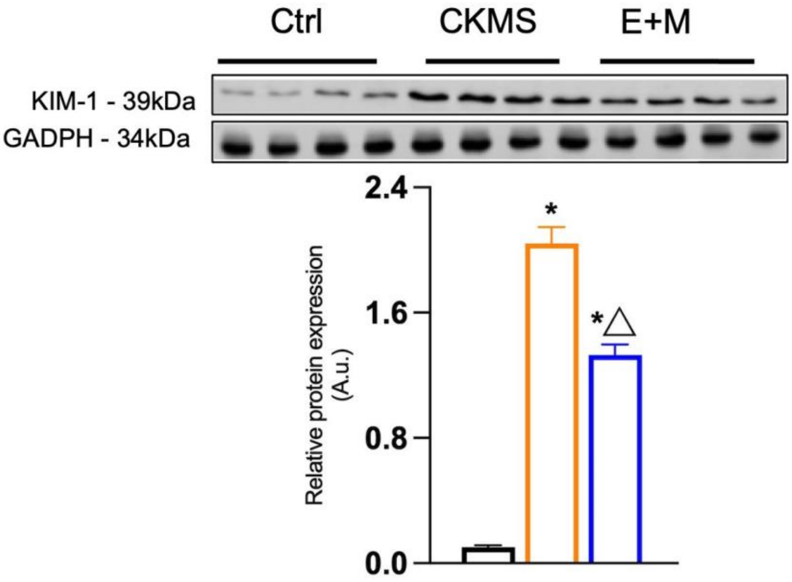

Figure S2. Effect of E+M combination treatment on renal tubular injury marker KIM-1. Representative immunoblot and densitometric analysis of KIM-1 protein relative expression normalized to GAPDH. Open bars in black lines (□) represent the control group (Ctrl), and open bars in orange lines (□) correspond to the cardio-kidney metabolic syndrome group (CKMS), and the bar in blue lines (□) represents the combination empagliflozin+metformin group (E+M). Data are expressed as mean ± SEM (n=4 biologically independent samples per group) and analyzed by one-way ANOVA with Tukey's multiple comparison tests. *p < 0.05 vs control group; △p < 0.05 vs CKMS group.

Third, as an indicator of renal epithelial tight junction integrity, we analyzed the protein expression of claudin-2 in kidney tissue homogenates. The control group displayed normal physiological levels of claudin-2 expression. In contrast, the CKMS group exhibited a significant decrease in claudin-2 protein levels (Supplementary figure 3, $p < 0.05$ vs Control group), indicating an impairment of tight junction proteins. Interestingly, combination treatment with E+M completely restored claudin-2 protein expression back to levels comparable to the control group (Supplementary figure 3, $p < 0.05$ vs CKMS group). These findings suggest that E+M combination therapy helps preserve renal epithelial barrier structural integrity in CKMS.

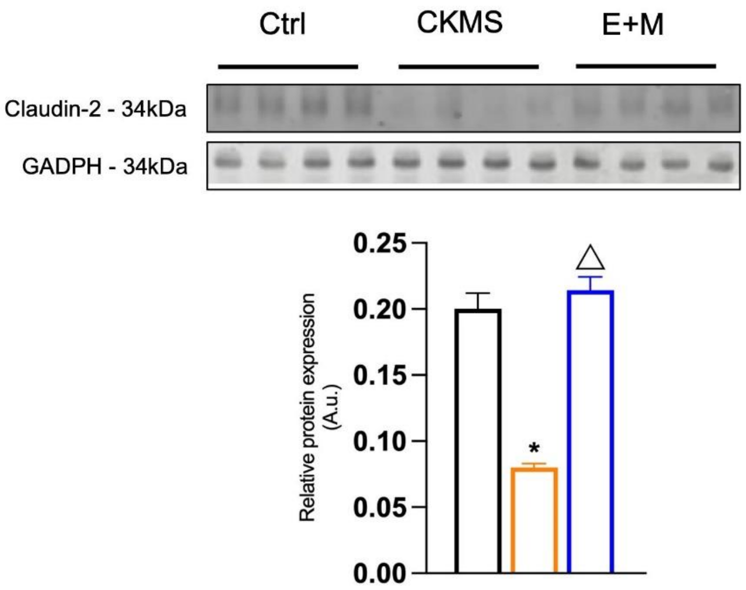

Figure S3. Effect of E+M combination treatment on renal tight junction protein claudin-2. Representative immunoblot (A) and densitometric quantification of claudin-2 protein relative expression normalized to GAPDH loading control. Open bars in black lines (□) represent the control group (Ctrl), and open bars in orange lines (□) correspond to the cardio-kidney metabolic syndrome group (CKMS), and the bar in blue lines (□) represents the combination empagliflozin+metformin group (E+M). Data are expressed as mean ± SEM (n=4 biologically independent samples per group) and analyzed by one-way ANOVA with Tukey's multiple comparison tests. *$p < 0.05$ vs control group; $^{\triangle}p < 0.05$ vs CKMS group.
